# Pharmacokinetics Modelling Reveals Inconsistency Between the US and European Vitamin D Safety Guidelines

**DOI:** 10.3390/biomedicines14091974

**Published:** 2026-09-01

**Authors:** Zhonghui Huang, Nadda Muhamad, Samantha Christie, Tao You

**Affiliations:** 1 Department of Pharmacology & Therapeutics, University of Liverpool, Liverpool L69 3GE, UK; zhonghui.huang@liverpool.ac.uk; 2 Centre of Excellence for Long-Acting Therapeutics (CELT), University of Liverpool, Liverpool L69 3GE, UK; 3 Department of Clinical Infection, Microbiology and Immunology, University of Liverpool, Liverpool L69 7ZX, UK; 4 Department of Pharmaceutical Care, Faculty of Pharmaceutical Sciences, Kasetsart University, Bangkok 10900, Thailand; nadda.m@ku.ac.th; 5 Greenwich Integrated Health, London SE10 8SR, UK; drsamchristie@gmail.com; 6 Beyond Consulting Ltd., 14 Tytherington Park Road, Macclesfield SK10 2EL, UK

**Keywords:** vitamin D, safe dose, children, adolescent, PBPK modelling, nonlinear mixed effects

## Abstract

**Background:** For vitamin D, the US National Academies of Sciences, Engineering, and Medicine (NASEM) suggest serum 25-hydroxyvitamin D (25(OH)D) >125 nmol/L is linked to potential toxicity. The European Food Safety Authority (EFSA) recommends that daily doses up to 2000 IU (50 µg) are safe for children aged 1–10 years, and 4000 IU (100 µg) for those aged 11 years and over. We evaluated the consistency between these two guidelines. **Methods:** We compiled accessible under 18’s clinical trial data for external validation. We tested published physiologically based pharmacokinetic (PBPK) models. We used simulations to explore the serum 25(OH)D pharmacokinetic profiles in children (6–10) and adolescents (11–17). We fitted a new model to the external validation data. **Results:** Our PBPK model, previously developed for healthy adults, made good predictions for the mean of the validation data. Model simulations suggested the mean should attain serum 25(OH)D > 115 nmol/L among 6-year-old children who take 2000 IU daily, and >125 nmol/L for 11- to 12-year-olds who take 4000 IU daily. We also presented clinical data to demonstrate that 4000 IU daily dosing increased serum 25(OH)D >125 nmol/L in a large proportion of healthy adults. Model parameters for the Cape Town children and the US and European children are different. **Conclusions:** This analysis highlights the inconsistency between the NASEM and EFSA guidelines and warrants the need for further pharmacokinetic modelling work to support the development of vitamin D safety parameters and guidelines. Our modelling suggests that the currently adopted 125 nmol/L threshold may warrant re-evaluation, as the supporting evidence appears heterogeneous and limited.

## 1. Introduction

The serum level of the vitamin D metabolite 25-hydroxyvitamin D (25(OH)D) is widely accepted as a marker for vitamin D status. In the US, according to the National Academies of Sciences, Engineering, and Medicine (NASEM) and the National Institute of Health (NIH), the working definition of vitamin D status includes deficiency (<30 nmol/L), inadequacy (30–50 nmol/L), adequacy (50–125 nmol/L), and potential toxicity (>125 nmol/L) [1,2]. For children and adolescents (ages 1–18 years), the Endocrine Society recommends vitamin D supplementation to prevent nutritional rickets and potentially lower the risk of respiratory tract infections and osteomalacia [3]. Daily doses between 300 and 2000 IU of vitamin D_3_ (“vitamin D” onwards) are assessed in studies on respiratory tract infection prevention, but no specific dose is recommended by the Endocrine Society [3]. According to the European Food Safety Authority (EFSA), a daily dose of vitamin D up to 2000 IU (50 µg) is safe for long-term use in children aged 1–10 years, and 4000 IU for anyone 11 or over [4]. This paper inspects the consistency between the NASEM and EFSA guidelines.

For drug development, pharmacokinetic-pharmacodynamic (PK-PD) modelling is widely adopted by regulatory agencies to assess the safety and effectiveness of dosage, and to inform medicine labelling. Here, we used physiologically based pharmacokinetic (PBPK) modelling to integrate accessible information in an unbiased manner. We studied whether the 2000 IU/day dose of vitamin D for children (6–10) and the 4000 IU/day dose for adolescents (11–17) would increase serum 25(OH)D over the 125 nmol/L threshold.

We have developed and validated PBPK models of serum 25(OH)D for orally administered vitamin D (Table 1). We first reported a naïve-pooled model, “Model 0” in Table 1, to predict the mean serum 25(OH)D from published clinical trials for healthy adults [5]. We used 56 trial arms to calibrate the model. It accurately predicted 16 other arms of large, bolus-style single doses (1250–50,000 µg) and 83 arms of daily doses (12.5–1250 µg) for validation [5] (Table 1).

We further developed this model into a nonlinear mixed effects (NLME) adult model, “Model 1” in Table 1, to capture the variability in mean serum 25(OH)D between arms [6] Table 1). The modelling identified weight as a crucial factor for accurate predictions. PK modelling of orally administered vitamin D was reported for children with chronic kidney disease [8] or with obesity and asthma [9]. Consistently, these reports also highlighted the importance of a weight-based approach to dose selection [8,9].

For external validation, this healthy adult model made accurate predictions for 7 additional clinical trial arms it had not seen before: 16 weeks at 250, 1250, and 2500 µg/day; 26 weeks at 250 µg/day; 152 weeks at 10, 100, and 250 µg/day [6]. As the test data include time courses ranging from 16 weeks to 3 years, this model was suitable for both short- and long-term vitamin D interventions.

To make long-term predictions for healthy children, we revised the healthy adult models [5,6] to fit individual-level data from a study of healthy schoolchildren in Cape Town, South Africa (baseline age 6–11 years) [10]. These include serum 25(OH)D in 77 children at baseline, 1, 2, and 3 years [7]. As a test, the model (“Model 2” in Table 1) accurately predicted the 3-year serum 25(OH)D measurements in 463 other children from the same study, with a mean estimation error of 5.3 nmol/L [7]. Covariates in this model included sex, weight, and the age-normalised body mass index Z score (ZBMI).

This paper inspects whether the 125 nmol/L serum 25(OH)D threshold and the EFSA recommended safe dose are consistent. We first tested Models 1 and 2 using European and US children’s data. We then simulated serum 25(OH)D PK at daily doses up to 2000 IU in children (6–10 years) and 4000 IU in adolescents (11–17 years). We evaluated two baseline levels, one representing severe deficiency (10 nmol/L baseline) and one for adequacy (50 nmol/L baseline). We also scrutinised the methodology EFSA and NASEM employed. To provide concrete evidence, we presented clinical serum PK in healthy adults who received a 4000 IU daily dose. We fitted a new model and inferred the clearance and disposition parameters for 25(OH)D among under 18’s in Europe and the US. Our work suggests the choice of thresholds for a safe serum 25(OH)D threshold needs to be informed by PK modelling. To gain insights, we envisage a model should be developed to consistently integrate PK, vitamin D receptor binding, calcium metabolism and clinical toxicity for this.

## 2. Materials and Methods

### 2.1. Construction of the Previous Models

**Figure 1:**
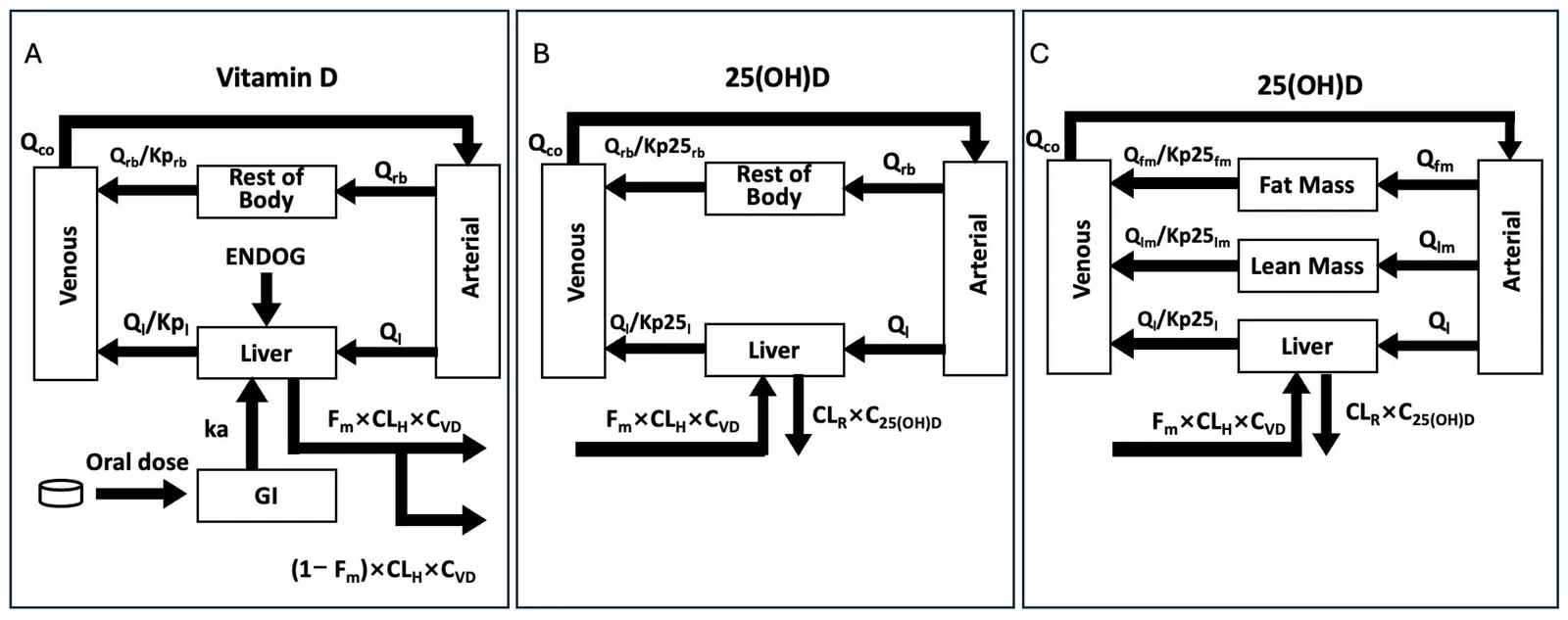
Model schematic diagram. (**A**) Vitamin D model. (**B**) 25(OH)D model for Model 1 (healthy adults) [6]. (**C**) 25(OH)D model for Model 2 (healthy children) [7].

The development and validation of the NLME PBPK models for healthy adults [6] and healthy children [7] were reported previously. Briefly, for healthy adults, orally dosed vitamin D was assumed to enter the gastrointestinal tract followed by the liver (Figure 1A). One in three of the vitamin D from de novo skin synthesis and food intake was assumed to be metabolised to 25(OH)D in the liver. All non-eliminating organs were combined to form a “the rest of the body” compartment (Figure 1A). This simplification was needed to ensure all parameters could be fitted from the observed vitamin D and 25(OH)D PK data. Weight was used to explain the variability of the volume for all compartments.

For healthy adults, 25(OH)D was modelled the same way as vitamin D (Figure 1B). In healthy children, the rest of the body compartment was further divided into fat mass and lean mass compartments for 25(OH)D (Figure 1C).

To select the best model, a series of nested models was assessed for the quality of parametric inference (uncertainty in fixed effects <20% and shrinkage in random effects <30%) and the goodness of fit (Akaike Information Criteria (AIC) and Bayesian Information Criteria (BIC)). The best model exhibited no patterns in residual errors and passed the visual predictive checks.

Model fitting was performed in R (v4.2.2) with the nlmixr2 R package (v2.0.9, SAEM routine) on a multi-core Linux cluster. Visual predictive check (VPC) was carried out using the vpcSim function from the nlmixr2est R package v2.1.5. Simulations were carried out with the rxode2 R package v2.0.13.

### 2.2. Compilation of External Validation Dataset

To further qualify these models, we found additional vitamin D trials in healthy subjects under 18 years in the United States and Europe. We searched Google Scholar and PubMed for serum 25(OH)D pharmacokinetics between 2005 and 2025, using the keywords “vitamin D supplementation” and “paediatrics” to find articles that provided 25-hydroxyvitamin D levels. The inclusion criteria were (1) written in English; (2) included healthy subjects under 18; (3) reported 25(OH)D at least at baseline and one follow-up; (4) study conducted in the USA or Europe. The exclusion criteria were (1) the study did not report WT or BMI; (2) did not report the dose; (3) subjects had conditions that affect vitamin D metabolism. Treatment regimen, dosage, age, sex, weight, height, BMI, or BMI-for-age Z score (ZBMI) were reported at baseline, while 25(OH)D concentration was measured at baseline and follow-up visits. Continuous variables were extracted as mean ± standard deviation or median with interquartile range, as reported in each study. Our Model 2 uses covariates including sex, weight, and ZBMI:

(1) $$ZBMI=\frac{{\left(\frac{BMI}{M}\right)}^{L}-1}{L\cdot S}$$

M Median BMI depending on age;
S Generalised coefficient of variation for the data;
L Power parameter needed to remove skewness in data.

These parameters were provided by the US Centers for Disease Control and Prevention. Mean age of the trial arm was used. Sex was assigned according to the most participants in each study.

Observed mean and SD were extracted directly when available. For studies that reported the median and interquartile range (IQR), the median was used to approximate the mean. SD was estimated by dividing the difference between the upper and lower quartiles by 1.35, assuming an approximately normal distribution, according to the Cochrane Handbook [11].

### 2.3. Model Validation

To test Model 1’s ability to predict the mean, fixed effects parameters were sampled based on the mean estimates and the standard errors (SE) reported in [6]. Inter-individual variability and residual errors (additive error standard deviation: 0.00074 nmol/L; proportional error standard deviation: 0.046) were ignored. For each arm, 1000 virtual subjects were simulated at the reported baseline weight to predict the mean 25(OH)D serum concentration with 95% prediction intervals. These were compared with the observed mean concentrations and the standard errors of the mean. Predictive performance was further quantified using the root mean square error (RMSE) and mean prediction error (MPE). RMSE was the square root of the mean squared residuals between model predictions and observations. The MPE was the arithmetic mean of the residuals across all observations.

To test if Models 1 and 2 may predict the 2.5th and 97.5th percentiles correctly, population simulation was performed. For $K_{p25rb}$ and ${CL}_{max}$, each virtual study draws a sample of the fixed effects (according to the reported mean and standard error [6]) and 1000 samples of the random effects (according to the variance-covariance matrix [6]). Residual errors were small and omitted. Baseline 25(OH)D serum concentration was assumed to conform to a normal distribution and was sampled for each virtual individual according to the reported mean and SD. Each virtual study predicts the mean, 2.5th and 97.5th percentiles. We generated 30 virtual studies to obtain the 95% confidence intervals for the predicted mean, 2.5th and 97.5th percentiles. For comparison, the observed mean serum 25(OH)D concentrations and the SD of the mean were used.

### 2.4. Fitting and Qualification of Model 3 to the External Validation Data

We fitted a “Model 3” to the external validation data. Model 3 shares the same structure as Model 0 and Model 1: all non-eliminating organs are grouped into “the rest of the body” compartment. Model 3 captures the inter-individual variability:

$${\mathrm{CL}}_{\max}=e^{T{\mathrm{CL}}_{\max}+\eta_{{\mathrm{CL}}_{\max}}}\;\times \;({\frac{{\mathrm{WT}}_{\mathrm{cur}}}{30})}^{0.75}$$

$$K_{p25rb}=e^{{\mathrm{TK}}_{p25rb}+\eta_{K_{p25rb}}}$$

Model 3 assumes the volume of each compartment is directly proportional to weight:

$${\mathrm{CL}}_{\max}=e^{T{\mathrm{CL}}_{\max}+\eta_{{\mathrm{CL}}_{\max}}}\;\times \;({\frac{\mathrm{WT}}{30})}^{0.75}$$

$$V_{\mathrm{art}}={\mathrm{TV}}_{\mathrm{art}}\;\times \;\frac{\mathrm{WT}}{30}$$

$$V_{\mathrm{ven}}={\mathrm{TV}}_{\mathrm{ven}}\;\times \frac{\mathrm{WT}}{30}$$

$$V_{\mathrm{rb}}={\;\mathrm{TV}}_{\mathrm{rb}}\;\times \;\frac{\mathrm{WT}}{30}$$

$$V_{l}={\mathrm{TV}}_{l}\times \;\frac{\mathrm{WT}}{30}$$

Essentially, Model 3 has the same covariate structure as the “fit 4” model from Table S5 in [7]. Model 3 shares the same physiological parameters as Model 2.

To qualify Model 3, we assessed the goodness of fit, conditional weighted residuals (CWRES) and normalized prediction distribution errors (NPDE), and visual predictive check. These were generated the same way as reported before [6].

### 2.5. Model Simulation of Cohorts with Baselines at 10 or 50 nmol/L

In a virtual study, for children aged from 6 to 10 years, weight was sampled according to the WHO report [12]. For adolescents aged from 11 to 17 years, height [13] and body mass index (BMI) [14] were sampled independently according to the WHO reports to calculate the weight. A virtual study contained 1250 boys and 1250 girls.

Random effects of the partition coefficient $K_{P25rb}$ (ratio between 25(OH)D concentration in the rest of the body and the serum) and CL_max_ (the maximum clearance rate constant) were sampled according to the variance-covariance matrix. Additive errors and proportional errors are small. They were ignored so as to start simulations from a specific baseline at either 10 or 50 nmol/L.

To explore the expected mean (Table 2), Model 1 was simulated to generate 30 virtual studies for each age group.

To estimate the 2.5th, 50th, and 97.5th percentiles, and the 95% prediction intervals for these percentiles (Figure S1), Model 3 was simulated to generate 30 virtual studies for each age group.

### 2.6. Compilation and Visualization of Healthy Adult Vitamin D Data

For healthy adults, we selected the 2000 IU (50 µg) and 4000 IU (100 µg) daily dosing arms from a previously compiled dataset [5]. The searching strategy was reported earlier [5], and the trials were reported between 2000 and 2021. Cubic polynomial regression was applied to describe the concentration-time profiles of all arms in a naïve pooled approach. We then generated 95% prediction intervals from the fitted polynomial model.

## 3. Results

### 3.1. Compilation of External Validation Data

**Figure 2:**
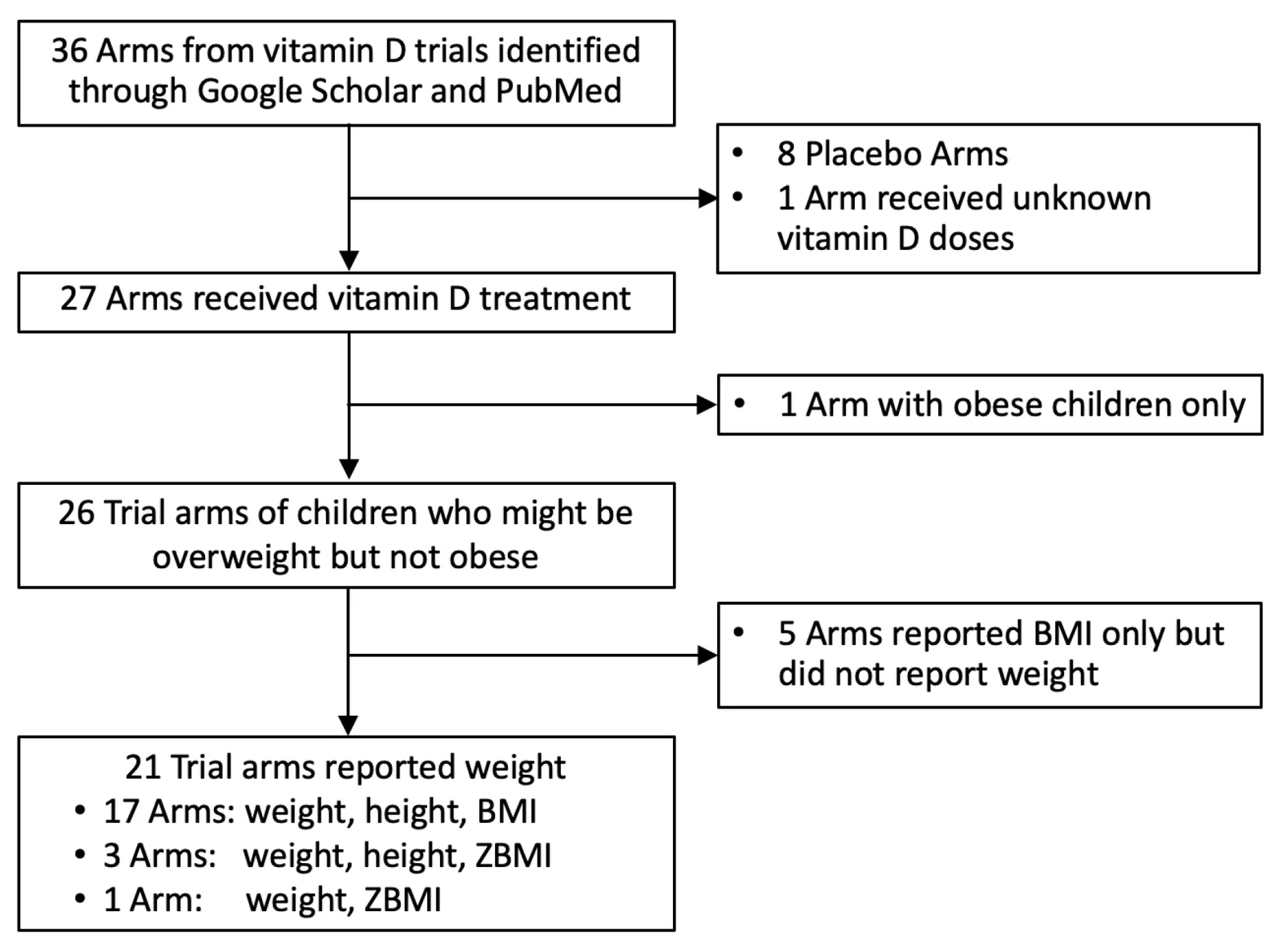
The selection of trial arms for external validation. All 21 trial arms selected are from healthy subjects who are under 18. They all reported age, weight, and percentage of sex.

Through literature searching, we identified 21 arms from 11 clinical trials, which reported serum 25(OH)D time course together with age, weight, and percentage of sex (Figure 2). The average age, body weight, and height were 11.5 ± 2.1 years, 46.9 ± 10.9 kg (Figure S2A), and 150.1 ± 33.7 cm, respectively. The average ZBMI was 0.87 ± 0.44 (Figure S2B). The average baseline 25(OH)D concentration was 54.7 ± 12.0 nmol/L. The vitamin D doses ranged from 200 to 4000 IU (5–100 µg) daily, with treatment durations from 1 month to 1 year. Baseline characteristics are provided in Table S1, and the references are provided in Table S2 [15,16,17,18,19,20,21,22,23,24,25].

### 3.2. Data Exploration

**Figure 3:**
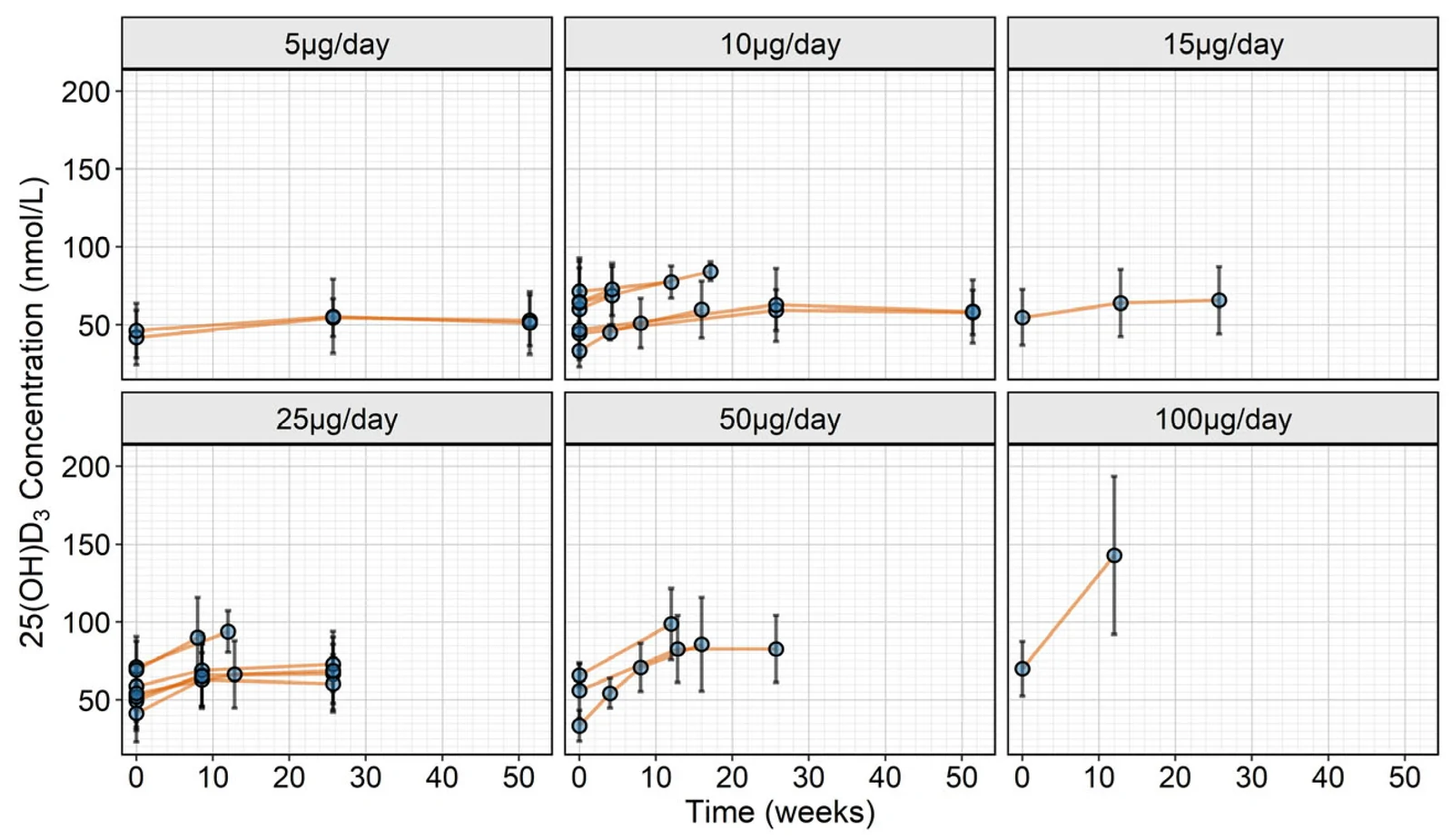
Serum 25(OH)D concentration profiles for external validation. Each panel shows a daily vitamin D dose. Points: mean serum 25(OH)D concentrations. Error bars: standard deviation.

The external validation data are stratified into six different doses (Figure 3). Serum 25(OH)D took at least 12 weeks to reach a pharmacokinetic steady state. This pattern was most obvious for subjects who received 10, 25, and 50 µg daily. Model 2 was developed to make annual predictions, and it was not calibrated to make predictions for periods less than a year. To qualify Model 2, we will compare its prediction at the end of year 1 with the last time point of each arm, which was observed at 12 weeks or later.

### 3.3. Model 1 Predicted the Mean but Overestimated the 2.5th Percentile

**Figure 4:**
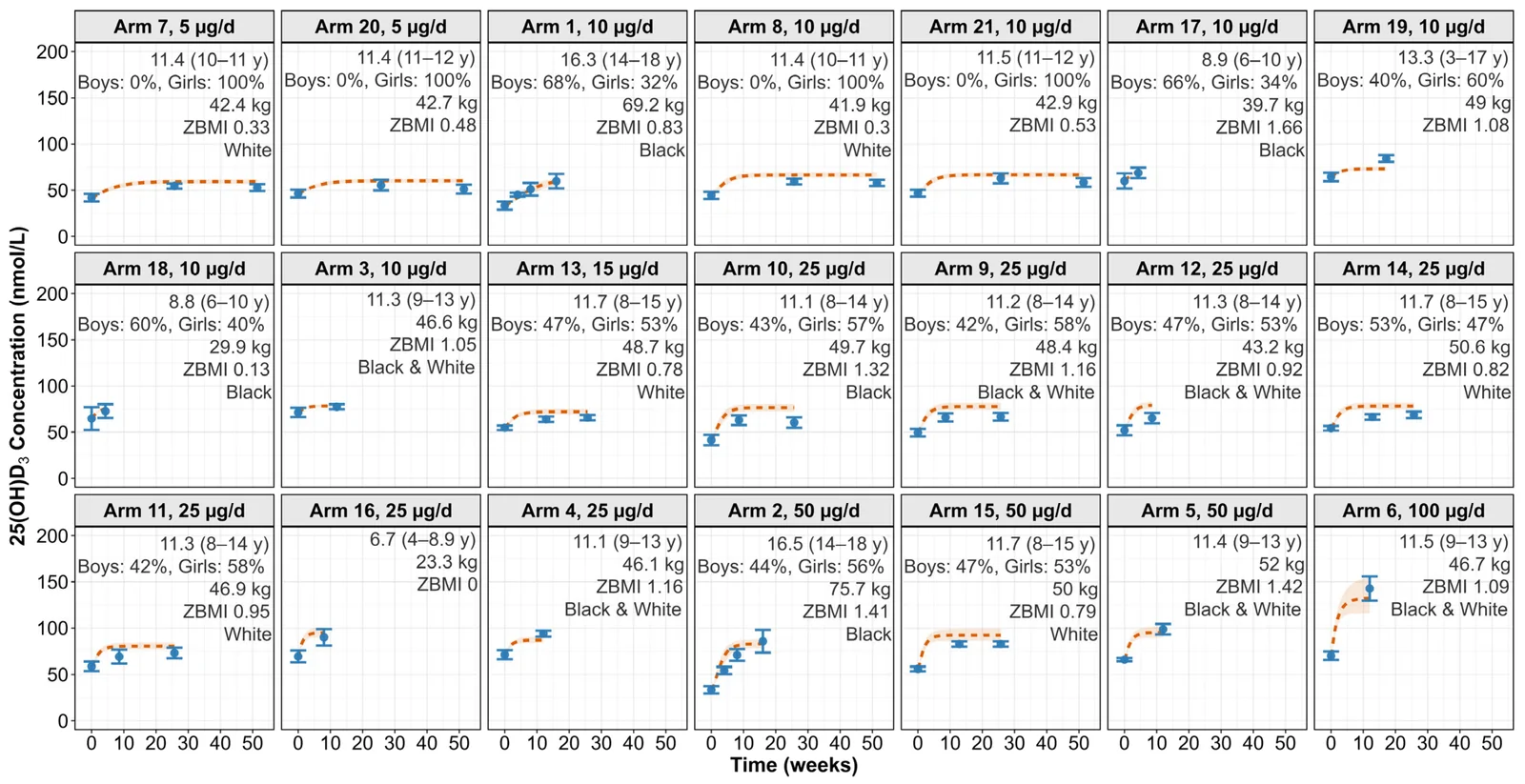
Predicted mean concentration profiles by Model 1. Each panel shows the results of 1000 virtual subjects. Blue dots: observed mean serum 25(OH)D. Error bars: standard error of the mean. Red dashed line: predicted mean. Red band: 95% prediction interval for the mean. Panels are sorted by the dose and the baseline 25(OH)D.

We simulated Model 1 to inspect whether it predicted the mean observations of each arm. We plotted the predicted mean (red lines) and its 95% prediction intervals (red bands) with the observations (mean: blue dots; standard error of the mean: error bars) (Figure 4). At 25 µg daily doses, the model overestimated the increase by more than 1 SE of the observations in 4 out of 6 cases (Arms 11, 12, 14 and 16). At other doses, model predictions were within ±1 SE of the observations. Across all observations, the RMSE and MPE were 6.8 nmol/L and 3.3 nmol/L, respectively. The residuals (i.e., observation—prediction) had a zero-mode estimated by the kernel density (Figure S3). The approx. 5% prediction bias was not associated with dose (Figure S4). Overall, model predictions were in good concordance with data.

**Figure 5:**
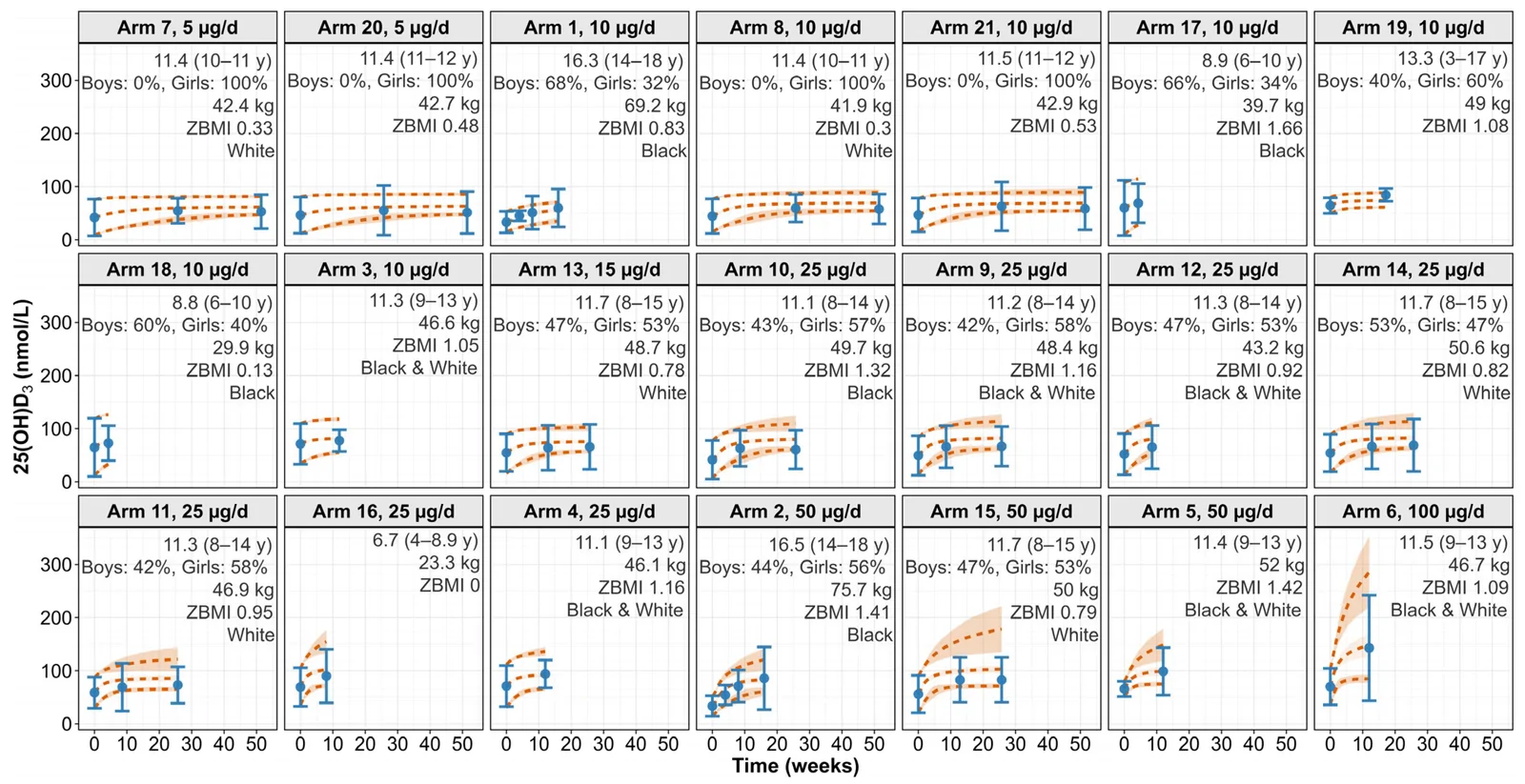
Predicted concentration profiles by Model 1. In each panel, 30 virtual studies were generated, with each virtual study containing 1000 subjects. Blue dots: observed mean serum 25(OH)D concentration. Error bars: 95% confidence interval of the observed mean. Central red dashed line and shades: predicted mean concentration with 95% prediction interval. Upper and lower red shades: 95% prediction interval of the predicted 97.5th and 2.5th percentiles, respectively.

We then inspected the 2.5th and 97.5th percentiles for model predictions. For each arm, we simulated from baseline 25(OH)D levels within the 95% confidence interval of the observations. The predicted 97.5th percentiles (upper red bands) are in good agreement with observations (error bars) except for Arm 17 (Figure 5). For the 2.5th percentile, Model 1 made good predictions for Arms 1, 5 and 23 at 10 µg/day, Arm 6 at 25 µg/day and Arm 2 at 50 µg/day (Figure 5). However, Model 1 overestimated the 2.5th percentiles in all other cases (Figure 5).

### 3.4. Model 2 Tended to Overestimate the Validation Data

**Figure 6:**
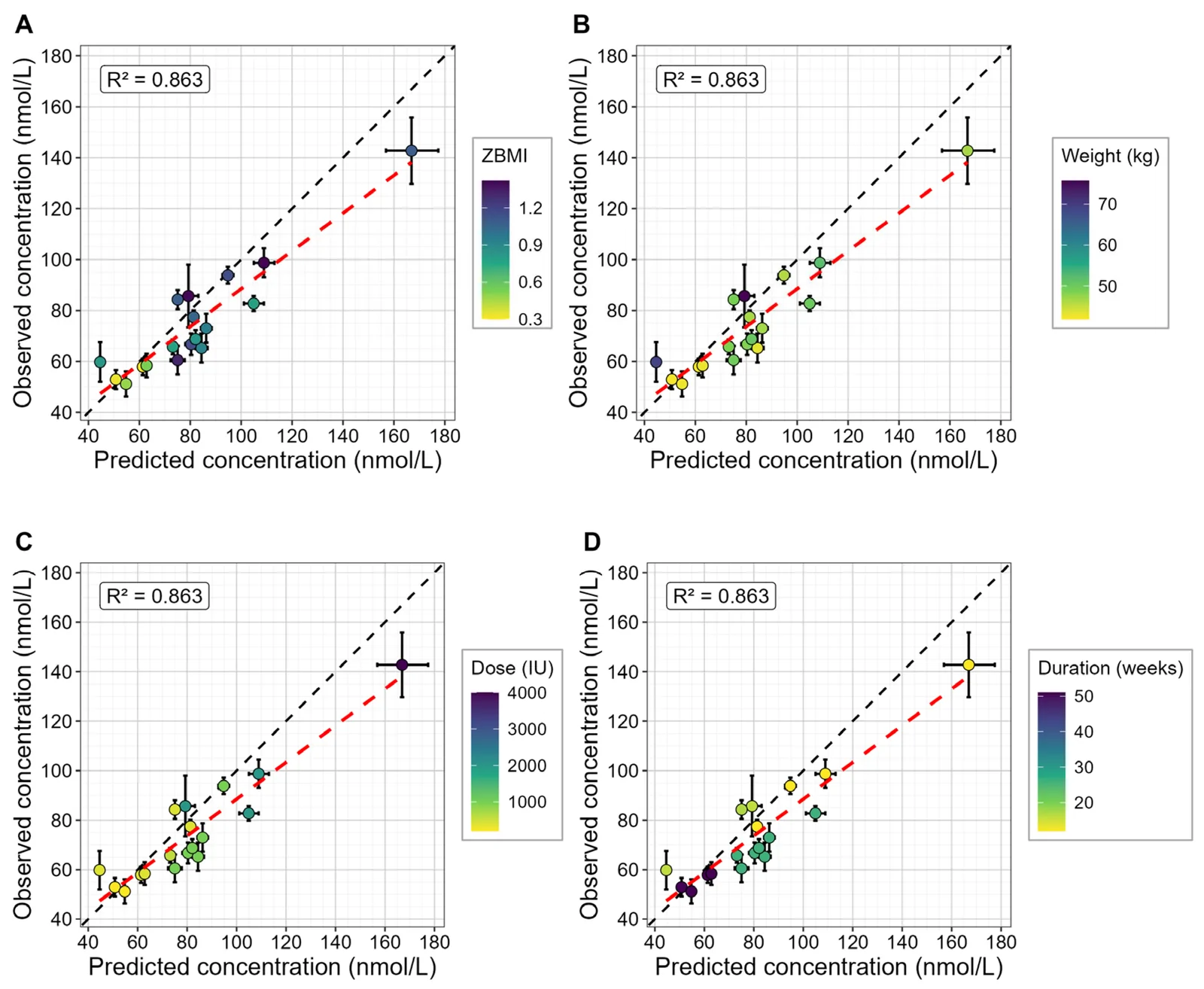
Observed serum 25(OH)D and Model 2 predictions. The filled circles are colour-coded with respect to ZMBI (**A**), weight (**B**), dose (**C**) and duration (**D**). Horizontal error bars: 95% prediction intervals for the mean. Vertical error bars: ±1.96 × standard error of the mean for observations. Some error bars are shorter than the symbols. Red dashed line: linear regression line.

As shown in Figure 3, it took at least 12 weeks to reach pharmacokinetics steady state. Model 2 was fitted to the annual data for a 3-year period [7]. Hence, it shall only be used to make annual predictions. To compare PK steady states, we selected the last time point taken at or after 12 weeks in each arm to compare with Model 2’s 1-year predicted mean. These exhibited a high degree of concordance with R^2^ = 0.86 (Figure 6). The RMSE and MPE were 12.41 nmol/L and 6.73 nmol/L, respectively.

There was a noticeable tendency for overprediction (see linear regression line in Figure 6). To investigate which clinical covariate may underpin the tendency, we stratified the model predictions by ZBMI, weight, dose, and the time point (i.e., duration) (Figure 6). ZBMI and duration did not have a clear influence on model predictions (Figure 6A,D). Prediction bias appeared to increase with dose (Figure 6C). However, the association was insignificant (*p* = 0.18 in Figure S6). Prediction bias was found to be significantly anticorrelated with weight (*p* = 0.034, Figure S7). Two arms with the highest mean weight (Arms 1 and 2) were underpredicted, in contrast to the overall overestimate trend (Figure 6B).

The residual distribution was unimodal with the mode of approximately −6 nmol/L (Figure S5). We fitted a “Model 3” to the external validation data (Section 4.5) to infer what might underlie the difference.

### 3.5. Model 1 Simulation Suggests Children Taking 4000 IU Vitamin D Daily May Exceed the 125 nmol/L Threshold

For simplicity, we simulated Model 1 to explore the mean PK. We simulated continuous daily dosing at 500, 1000, and 2000 IU in 2500 virtual subjects between 6–17 years and 4000 IU in 2500 virtual subjects between 11–17 years (Table 2). Each simulation contained 1250 boys and 1250 girls. The 95% prediction intervals for the 50th percentiles are small. Hence, we focus on the predicted mean for the 50th percentile. This is stratified by baseline age and baseline serum 25(OH)D (Table 2). Older age is associated with heavier weight and larger volume of distribution. Consistently, the magnitude of change in serum 25(OH)D decreases with age (see each column of Table 2).

Among the 11-to 12-year-olds receiving 4000 IU daily, the 50th percentile was predicted to exceed 125 nmol/L, irrespective of the baseline 25(OH)D (grey in Table 2). This highlights inconsistency between the EFSA and NASEM guidelines. Next, we will probe where the difference may come from.

## 4. Discussion

Here, we will first discuss how the NASEM 125 nmol/L threshold and the EFSA safe upper level were developed. We will then look into the observed serum 25(OH)D in adults receiving 2000 IU and 4000 IU vitamin D daily. These data also highlight the inconsistency between the two guidelines. We will fit a new model to the external validation data and compare the parameters with Model 2. Finally, we will discuss what types of pharmacometrics modelling may help quantitatively understand vitamin D safety and inform the selection of a safe dose.

### 4.1. Derivation of the EFSA Safe Upper Level (2000 IU/day)

To derive the daily safe upper level (UL) of vitamin D for children, EFSA first identified the lowest observed adverse effect level (LOAEL) from two randomized controlled trials of healthy adults, which was 10,000 IU/day [4]. EFSA then applied a 2.5-fold uncertainty factor to obtain the UL of the safe daily dose of 4000 IU for anyone aged over 11 years [4]. For children aged 1–10 years, a 0.5-fold factor was applied to obtain the daily UL at 2000 IU [4].

### 4.2. Derivation of the NASEM 125 nmol/L Safety Threshold

The NASEM 2011 report [1] discussed the origin of the 125 nmol/L safety threshold in the context of cardiovascular risk and the upper limit of safe intake for adults. They are discussed below.

#### 4.2.1. Cardiovascular Risk

On page 417, the NASEM report claimed an observational study by Melamed et al. [26] found a “lower risk of CVD mortality in men and women at levels of 75 to 122 nmol/L, but a higher risk of CVD mortality in women at levels above 125 nmol/L” [1]. In fact, that study did not make such a claim [26]. Instead, that article only reported that low serum 25(OH)D was associated with the prevalence of peripheral arterial disease (PAD) and did not discuss 125 nmol/L at all [26]. For each 10 ng/mL lower 25(OH)D level, the multivariable-adjusted prevalence ratio of PAD was 1.35 (95% confidence interval: 1.15, 1.59) [26].

The NASEM report also cited another study of adults aged 65 years and older as evidence that high serum 25(OH)D is associated with elevated cardiovascular risk [27]. On page 437, the report reads, “analysis of fully adjusted data indicated an inverse relationship between CVD mortality and baseline serum 25(OH)D level of 50.0 to 74.9 nmol/L. Risk began to increase at approximately 75 nmol/L, and then it declined after 100 nmol/L. In fact, the original article suggested that serum 25(OH)D levels had a strong linear inverse association with mortality risk (hazard ratio = 0.95, 95% CI = 0.92–0.98 for every 10-nmol/L increase in 25(OH)D) [27]. This is different from the NASEM report.

It is not possible to know why the NASEM report made different interpretations from those made by the original studies. Relevant to this, a 2019 meta-analysis of over 83,000 individuals in 21 randomized controlled trials reported vitamin D supplementation was not associated with reduced major adverse cardiovascular events, individual CVD end points, or all-cause mortality [28]. Mean baseline serum 25(OH)D ranged between 7 ng/mL (17.5 nmol/L) and 53 ng/mL (132.5 nmol/L) [28]. These findings do not readily support the idea that vitamin D supplementation may confer cardiovascular protection and are not indicated for this purpose [28]. Some of these trials may have drawbacks in their design and execution. A critical appraisal of large vitamin D RCTs was given recently [29]. An analysis gave tantalizing results for CVD prevention [30]. Indeed, the role of vitamin D in disease prevention was reviewed [31].

#### 4.2.2. Safe Upper Level of Vitamin D Intake for Ages 19 or Older

The NASEM 2011 report selected an intake of 10,000 IU/day as NOAEL (page 443 in [1]). As with the EFSA, they applied a 2.5-fold safety factor to obtain an upper safe level of intake at 4000 IU/day (page 443 in [1]).

Interestingly, on the same page, the report initially recognized that serum 25(OH)D levels given maximal sun exposure, remain below 150 nmol/L, then claimed that emerging data related to all-cause mortality, chronic disease risk, and falls suggest adverse events may occur with serum 25(OH)D levels of approximately 75 nmol/L or above, and finally stated that 125–150 nmol/L might be the range for toxicity (pages 443–444 of [1]). Here, the report once again cited the two CVD references as discussed above [26,27], as well as a study of risk of nursing home admission among the elderly [32], which specifically stated people with high 25(OH)D (>75 nmol/L) had the lowest risk of nursing home admission [32]. Once again, the NASEM report made an entirely different interpretation of the original studies.

### 4.3. Other References to the 125 nmol/L Serum 25(OH)D in the Context of Vitamin D Deficiency (NASEM 2011 Report)

#### 4.3.1. Serum Parathyroid Hormone (PTH) Levels to Define Vitamin D Deficiency

Vitamin D deficiency reduces calcium absorption and serum calcium levels. In response, the calcium-sensing receptor upregulates the synthesis of PTH to release mineral from bone and to increase calcitriol (1,25(OH)_2_D) synthesis. High PTH may reduce serum phosphate, disrupt calcium intake, and cause nutritional rickets [33].

Numerous studies have explored whether an increase in PTH levels may be used to identify a cutoff value for serum 25(OH)D, a prognostic marker for vitamin D deficiency, and reported a large range of cutoff values for serum 25(OH)D, spanning from <30 nmol/L to <125 nmol/L; see Box 4–7 on page 261 of the NASEM report [1]. As an extreme example, Dawson-Hughes and colleagues reported marginal reduction in serum PTH among healthy elderly with 25(OH)D over 125 nmol/L (men: 3.24 ± 0.88 pmol/L; women: 3.56 ± 1.17 pmol/L) compared with those with 101–125 nmol/L serum 25(OH)D (men: 3.42 ± 1.75 pmol/L; women: 3.27 ± 1.42 pmol/L), suggesting the cutoff value might be much higher than 125 nmol/L [34]. These certainly contradict the conclusion that serum 25(OH)D > 125 nmol/L is harmful. More recent data also demonstrate that vitamin D and parathyroid hormone vary seasonally [35].

#### 4.3.2. Calcium Absorption

Using a single calcium isotope method, a study detected no difference for calcium absorption between participants with lower (approximately 75 nmol/L) and higher (approximately 125 nmol/L) serum 25OHD levels [36].

Overall, throughout the NASEM 2011 report, no robust evidence was presented to support the 125 nmol/L safety threshold. Hence, we suggest placing less emphasis on the 125 nmol/L threshold.

### 4.4. Serum 25(OH)D Concentration in Adults Receiving 2000 IU (50 µg) or 4000 IU (100 µg) Vitamin D Daily

**Figure 7:**
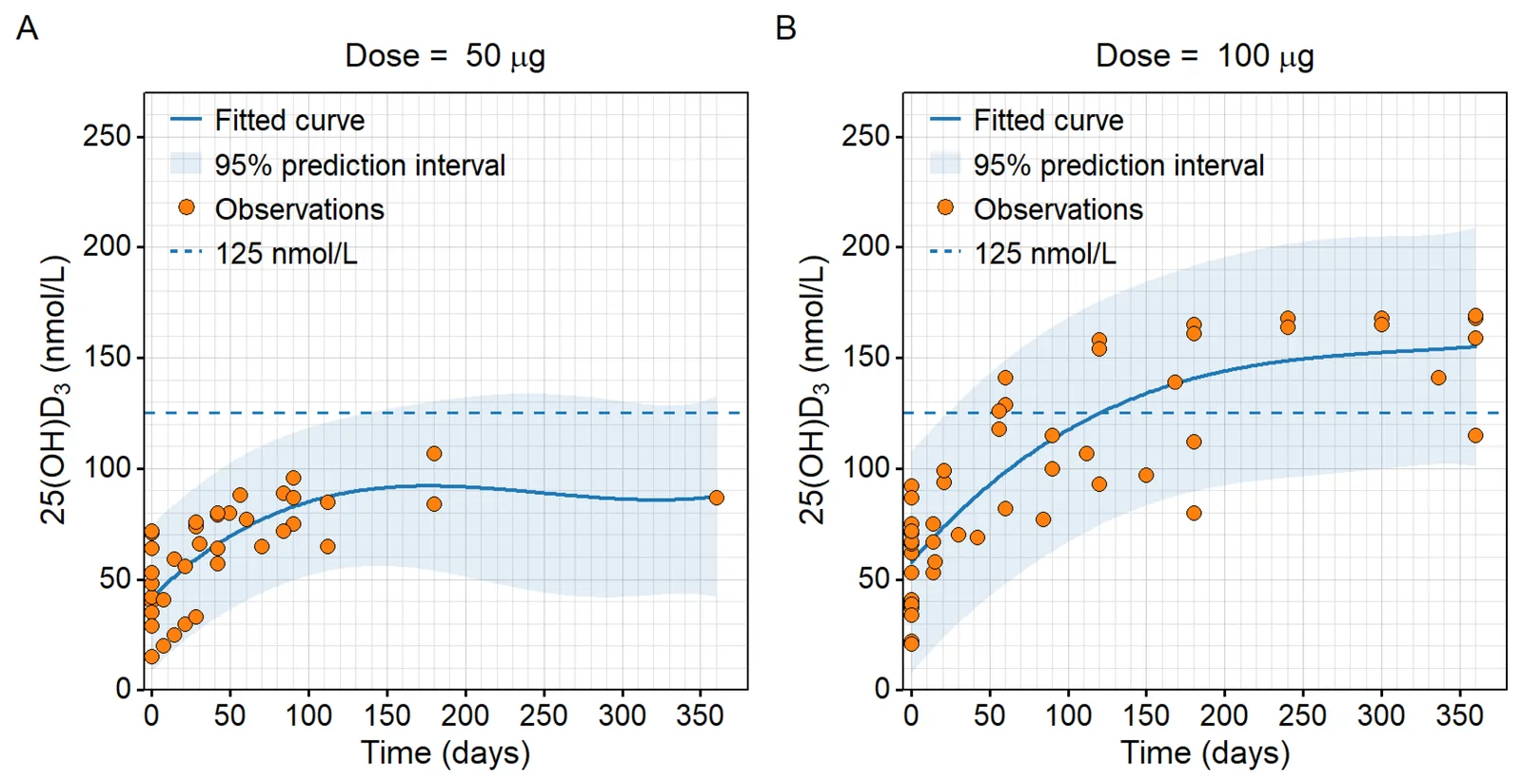
Serum 25(OH)D concentration–time profiles for oral daily doses at 2000 IU (50 µg) (**A**) and 4000 IU (100 µg) (**B**). Orange circles: observations. Solid blue lines: predicted mean of the 2nd-order polynomial equation. Shaded area: 95% prediction intervals. Dashed line: 125 nmol/L.

For adults who received daily doses of 2000 IU and 4000 IU, we identified reported data (Table S3; 2000 IU (50 µg) [37,38,39,40,41,42,43,44,45,46,47,48] and 4000 IU (100 µg) in healthy adults [39,40,48,49,50,51,52,53,54,55,56]) and plotted the mean serum 25(OH)D in red dots in Figure 7. No mean values were reported to exceed 125 nmol/L at 2000 IU/day (Figure 7A). At 4000 IU/day, many observed mean values exceeded 125 nmol/L (Figure 7B). To summarise the mean observations, we fitted the data using a polynomial equation in a naïve pooled approach and sampled the 95% prediction intervals of the fitted equations. To be conservative, we should focus on the predicted mean by the polynomial equations. The mean predictions for the 4000 IU group reached 125 nmol/L at about 130 days (blue solid line in Figure 7B). At 1 year, the predicted mean was over 150 nmol/L. These highlighted the inconsistency between the two guidelines at 4000 IU/day for healthy adults.

### 4.5. Serum 25(OH)D Concentration in Cases of Vitamin D Toxicity

Exceeding a serum 25(OH)D concentration threshold alone does not necessarily imply toxicity. Serum 25(OH)D concentrations that were associated with clinical toxicity were extensively studied and reported from the 1970s to the 1990s [57]. Hypercalcemia due to vitamin D intoxication was associated with serum 25(OH)D concentration > 220 nmol/L [57], and in many cases >1000 nmol/L. The lowest dose associated with toxicity (osteomalacia) was 7500 µg (300,000 IU) once monthly for 90 months [58]. In this case, the final 25(OH)D was at 608 nmol/L. The same study also reported the lowest toxic 25(OH)D concentration (221 nmol/L) was associated with osteoporosis among subjects who administered 15,000 µg (600,000 IU) vitamin D daily for 96 weeks [58]. The 7500 µg once monthly dose is equivalent to the total amount of 250 µg daily (matching EFSA’s lowest-observed-adverse-effect-level [4]) for 30 days. The lowest serum 25(OH)D concentration associated with toxicity (221 nmol/L) far exceeded NASEM’s 125 nmol/L threshold. However, supplemental vitamin D doses of 3200–4000 IU/day appear to increase the risk of hypercalcemia and some other adverse events in a small proportion of individuals [59].

### 4.6. What Underlies the Difference Between Model 2 and External Validation Data?

The Mongolian ViDiKids study assessed serum 25(OH)D at baseline and the end of the 3 years [60]. Model 2 overestimated the serum 25(OH)D in Mongolian children [7]. It was not possible to fit a model due to the lack of time course [7].

Weight and ZBMI are different between the Cape Town children and the external validation data. The average weight was 30 kg for Cape Town children (baseline age: 6–11) (Figure S13 in [7]) and was 44 kg for the external validation data (baseline age: 6.7–16.5) (Figure S2A). The average ZBMI was 0 for the Cape Town children, the same as the WHO expectation [10] (also see Figure S6 in [7]). ZBMI was approximately 1 for the external validation data. This is 1 standard deviation above the WHO expectation (Figure S2B).

How do we explain the difference between Model 2 predictions and the external validation data? The maximum elimination rate constant ${CL}_{max}$ and the partition coefficient for all non-eliminating organs ${Kp}_{25rb}$ have opposing effects on the serum 25(OH)D. It is not immediately intuitive which parameter should be changed by how much to account for the difference between Model 2 and the external data. We fitted a Model 3 to the external validation data. For simplicity, Model 3 ignores weight changes and adopts the “the rest of the body” compartment as Models 0 and 1 (Figure 1A,B). Model 3 has the same physiological parameters as Model 2.

In Model 3, we fitted the external validation data well (R^2^ = 0.96 for population predictions, Figure S9A,B), and there was no pattern in the residuals (Figure S9C–F). The visual predictive check confirmed Model 3 could satisfactorily predict these data (Figure S10). Model 3 parameters were close to Model 1 and were distinct from Model 2 (Table S4). In other words, the characteristics of metabolism and disposition of 25(OH)D in the US and European children (${Kp}_{25rb}=0.30$, ${CL}_{max}=0.036L/h$ for a 30 kg child) may resemble the healthy adults (${Kp}_{25rb}=0.28$, ${CL}_{max}=0.046L/h$ for a 70 kg adult) and may be different from the Cape Town children (${Kp}_{25fm}=4.7$, ${CL}_{max}=0.012L/h$ for a 30 kg child). Yet, per kg of weight, ${CL}_{max}$ was higher in European and US children than in adults. How sure are we about Model 3 parameters? At nominal values, we varied ${CL}_{max}$ and ${Kp}_{25rb}$ singly from 0.01- to 10-fold (Figure S8). The RMSEs were mostly sensitive to the reduction in ${CL}_{max}$ and the increase in ${Kp}_{25rb}$. In other words, to agree with the data, the model suggests ${CL}_{max}$ should not be lower than 0.0463L/h, and ${Kp}_{25fm}$ should not be higher than 0.296. This suggests the difference between the Cape Town children and the European and US children was not merely due to weight and ZBMI.

The higher clearance for Model 3 suggests 25(OH)D might be eliminated faster in the US and European children than the Cape Town children. Darker skin is less efficient in producing vitamin D. Lower clearance rate may preserve vitamin D. In Cape Town children, $K_{p}$ is 4 for the lean mass and 4.7 for the fat mass, indicating 25(OH)D may preferentially distribute in the tissue than in the blood. When we reported this finding, we speculated this might be due to higher receptor binding in the tissue in children to meet the rapid growth demand [6,7]. However, $K_{p}$ in Model 3 is close to the adult values in Models 0 and 1. This warrants further investigation.

### 4.7. Future Pharmacometrics Modelling Work

Using simulation, we explored the inconsistency between EFSA and NASEM guidelines. The work in this paper is limited to the discussion of dose-PK relationship only, and PK exceedance does not necessarily imply harm. EFSA defines tolerable upper intake levels, and NASEM discusses serum concentration thresholds. These are related but not identical concepts. Intake recommendations and serum thresholds are distinct frameworks. Apparent discrepancies may partly arise from methodological differences rather than true contradiction.

The concentration of 25(OH)D that displaces 50% radiolabel from the vitamin D receptor in vitro is 138 nmol/L [61]. It is tempting to put a serum 25(OH)D threshold for safety. As vitamin D promotes the absorption of calcium at multiple steps, it is the interplay of vitamin D and calcium doses that determines serum calcium status [61]. Therefore, the inconsistency also highlights the need for additional modelling work to consistently integrate all accessible vitamin D data, preferably collected at the individual level, and preferably include the dosage (of vitamin D, its analogues and metabolites), 25(OH)D levels, calcium intake, and demographic information (at least sex, age, weight and race).

Ultimately, the goal should be to inform the selection of a vitamin D dose in the context of calcium intake, which is safe for the public. This requires a good understanding of the dose-adverse drug reaction (ADR) relationship. The model should consider clinical endpoints such as hypercalcemia, nephrocalcinosis, or other types of adverse reactions.

An ordinal regression model, also known as a proportional odds logistic regression model, is typically constructed to synthesize the link between dose, PK, and the occurrence of ADRs [62]. It may be adopted for vitamin D research, so that we can develop a rigorous, quantitative understanding of its safety. For instance, an ordinal model shall establish the link between PK and ADRs, and it may be used in conjunction with a PK model to relate ADRs with dose.

## 5. Conclusions

In summary, our modelling work uncovered an inconsistency between EFSA recommended safe doses and the NASEM’s proposed 125 nmol/L serum 25(OH)D threshold for vitamin D safety, which warrants further pharmacometrics modelling work to reconcile such differences. We scrutinized the NASEM report. We did not find direct evidence to support the claim that 125 nmol/L should mark toxicity.

## Figures and Tables

**Table 1:** Summary of three previously reported PBPK models of vitamin D_3_ oral doses.

	Model 0 [5]	Model 1 [6]	Model 2 [7]
Type	Naïve-pooled Mean data for each trial arm	NLME Mean data for each trial arm	NLME Individual level data
Fitting subjects	Healthy adults age: 18 and over	Healthy adults age: 18 and over	Healthy Cape Town children baseline age: 6–11 monitored for 3 years
Test subjects	Healthy adults age: 18 and over	Healthy adults age: 18 and over	Healthy Cape Town children baseline age: 6–11 monitored for 3 years
Fitting data	Vitamin D: 13 arms (single dose from 70 to 2500 µg up to 5 days; 20 to 275 µg QD up to 120 days) 25(OH)D: 43 arms (10 and 100 µg QD up to 1 year)	Vitamin D: 13 arms (single dose from 70 to 2500 µg up to 5 days; 20 to 275 µg QD up to 120 days) 25(OH)D: 90 arms (10 and 1250 µg QD up to 1 year)	25(OH)D: 77 children at 250 µg QW Measured at baseline, 1, 2 and 3 years
Test data	25(OH)D: 16 arms single dose from 1250 to 50,000 µg up to 270 days; 83 arms daily dose from 12.5 to 1250 µg QD up to 1 year	25(OH)D: 5 arms from 10 to 250 µg QD up to 3 years and 2 trial arms at 1250 and 2500 µg QW within 16 weeks	25(OH)D: 463 children at 250 µg QW (9 followed up at 2 years; 454 followed up at 3 years)
Continents	Asia, Americas, Europe, Oceania	Asia, Americas, Europe, Oceania	Africa
Assumptions	Baseline vitamin D production is different for each arm * All other parameters are the same	Baseline vitamin D intake, maximum clearance rate and partition coefficient are different for each arm * All other parameters are the same	Baseline vitamin D intake, maximum clearance rate and partition coefficient are different for each person * All other parameters are the same
Assumed parameters	$K_{pl}=1$ $F_{m}=0.33$	$K_{pl}=1$ $F_{m}=0.33$	$K_{pl}=1$ $F_{m}=0.33$
Random effects	-	${CL}_{max}=e^{{TCL}_{max}+\eta_{{CL}_{max}}}\times (\frac{WT}{70}{)}^{0.75}$ $K_{p25rb}=e^{{TK}_{p25rb}+\eta_{K_{p25rb}}}$	${CL}_{max}=e^{{TCL}_{max}+\eta_{{CL}_{max}}}\times (\frac{WT}{70}{)}^{0.75}$ $K_{p25rb}=e^{{TK}_{p25rb}+\eta_{K_{p25rb}}}$
Covariates	-	Weight	Weight, age, sex, ZBMI

* Baseline VD production includes de novo synthesis by skin and vitamin D from food intake. NLME: nonlinear mixed effects modelling. QD: once daily dosing. QW: once weekly dosing. ZBMI: Z score for age-normalised body mass index.

**Table 2:** The mean of the predicted 50th percentiles after 1-year dosing. Green: 50–125 nmol/L. Gray: >125 nmol/L. Model 1 was simulated.

	10 nmol/L Baseline				50 nmol/L Baseline			
Baseline Age	500 IU (12.5 µg)	1000 IU (25 µg)	2000 IU (50 µg)	4000 IU (100 µg)	500 IU (12.5 µg)	1000 IU (25 µg)	2000 IU (50 µg)	4000 IU (100 µg)
6	76	89	116	-	77	90	117	-
8	73	85	107	-	75	87	109	-
9	72	84	104	-	74	85	105	-
10	71	82	100	-	72	83	102	-
11	69	80	97	136	71	82	98	140
12	68	79	94	129	70	80	96	131
14	66	76	90	117	68	77	91	118
17	64	74	86	109	67	75	88	111

## Data Availability

Anonymized data from the ViDiKids Cape Town Trial may be requested from Professor Adrian Martineau to be shared subject to terms of research ethics committee approval.

## Supplementary Materials

The following supporting information can be downloaded at: https://www.mdpi.com/article/10.3390/biomedicines14091974/s1, Figure S1: Simulation of serum 25(OH)D concentration for 1-year continuous daily dosing in the US and European children; Figure S2: Distribution of weight (A) and ZBMI (B) in the external validation data; Figure S3: Residual distribution for the external validation of Model 1; Figure S4: Prediction bias of Model 1 across doses; Figure S5: Residual distribution for the external validation of Model 2; Figure S6: Prediction bias of Model 2 across doses; Figure S7: Prediction bias of Model 2 across body weights; Figure S8: Sensitivity analysis of Model 3 performance to changes in CL_max_ and Kp_25rb_; Figure S9: Diagnostic plots for Model 3; Figure S10: Visual predictive check for Model 3 fitting; Table S1: Baseline characteristics of the external validation dataset; Table S2: References for the external validation dataset Table S3: References for daily vitamin D doses of 2000 IU (50 µg) and 4000 IU (100 µg) in healthy adults; Table S4: Model parameters.

## Abbreviations

The following abbreviations are used in this manuscript:

η	Between individual variation
25(OH)D	25-hydroxyvitamin D
AIC	Akaike information criterion
BIC	Bayesian information criterion
BMI	Body mass index
CI	Confident interval
CL	Clearance
CWRES	Conditional weighted residuals
DAE	Differential-algebraic equations
EFSA	European Food Safety Authority
IOM	Institute of Medicine (US)
IU	International unit
K_p_	Partition coefficients
NASEM	National Academies of Sciences, Engineering, and Medicine
NLME	Nonlinear mixed-effects
NPDE	Normalised prediction distribution errors
ODE	Ordinary differential equations
PBPK	Physiologically based pharmacokinetic
PK	Pharmacokinetic
SAEM	Stochastic approximation expectation-maximisation
V	Volume of distribution
VPC	Visual predictive check
WT	Weight
ZBMI	BMI-for-age Z-score
