# Supplementary material for: Pharmacokinetics Modelling Reveals Inconsistency Between the US and European Vitamin D Safety Guidelines
Source: Biomedicines. 2026 Sep 1;14(9):1974. doi: 10.3390/biomedicines14091974 (PMC13604647; doi:10.3390/biomedicines14091974)
Supplement: Supplementary file 1 [file biomedicines-14-01974-s001.zip › biomedicines-4318867-supplementary.pdf]

Pharmacokinetics Modelling Reveals Inconsistency Between the US and European Vitamin D Safety Guidelines

Zhonghui Huang [1,2,3], Nadda Muhamad [4], Samantha Christie [5] and Tao You [1,2,6,*]

1 Department of Pharmacology & Therapeutics, University of Liverpool, Liverpool L69 3GE, UK; zhonghui.huang@liverpool.ac.uk
2 Centre of Excellence for Long-Acting Therapeutics (CELT), University of Liverpool, Liverpool L69 3GE, UK
3 Department of Clinical Infection, Microbiology and Immunology, University of Liverpool, Liverpool L69 7ZX, UK
4 Department of Pharmaceutical Care, Faculty of Pharmaceutical Sciences, Kasetsart University, Bangkok 10900, Thailand; nadda.m@ku.ac.th
5 Greenwich Integrated Health, London SE10 8SR, UK; drsamchristie@gmail.com
6 Beyond Consulting Ltd., 14 Tytherington Park Road, Macclesfield SK10 2EL, UK
* Correspondence: tao.you@liverpool.ac.uk

Supplementary Materials

Figure S1. Simulation of serum 25(OH)D concentration for 1-year continuous daily dosing in the US and European children. 4
Figure S2. Distribution of weight and ZBMI in the external validation data. 5
Figure S3. Residual distribution for the external validation of Model 1. 6
Figure S4. Prediction bias for Model 1 across doses. 6
Figure S5. Residual distribution for the external validation of Model 2. 7
Figure S6. Prediction bias of Model 2 across doses. 7
Figure S7. Prediction bias of Model 2 across body weights. 8
Figure S8. Sensitivity analysis of Model 3 performance to changes in CL_{max} and Kp_{25rb}. 8
Figure S9. Diagnostic plots for Model 3. 9
Figure S10. Visual predictive checks for Model 3 fitting. 10
Table S1. Baseline characteristics of the external validation dataset.. 11
Table S2. References for the external validation dataset. 12
Table S3. References for daily vitamin D doses of 2000 IU (50 µg) and 4000 IU (100 µg) in healthy adults 13
Table S4. Model parameters. 15

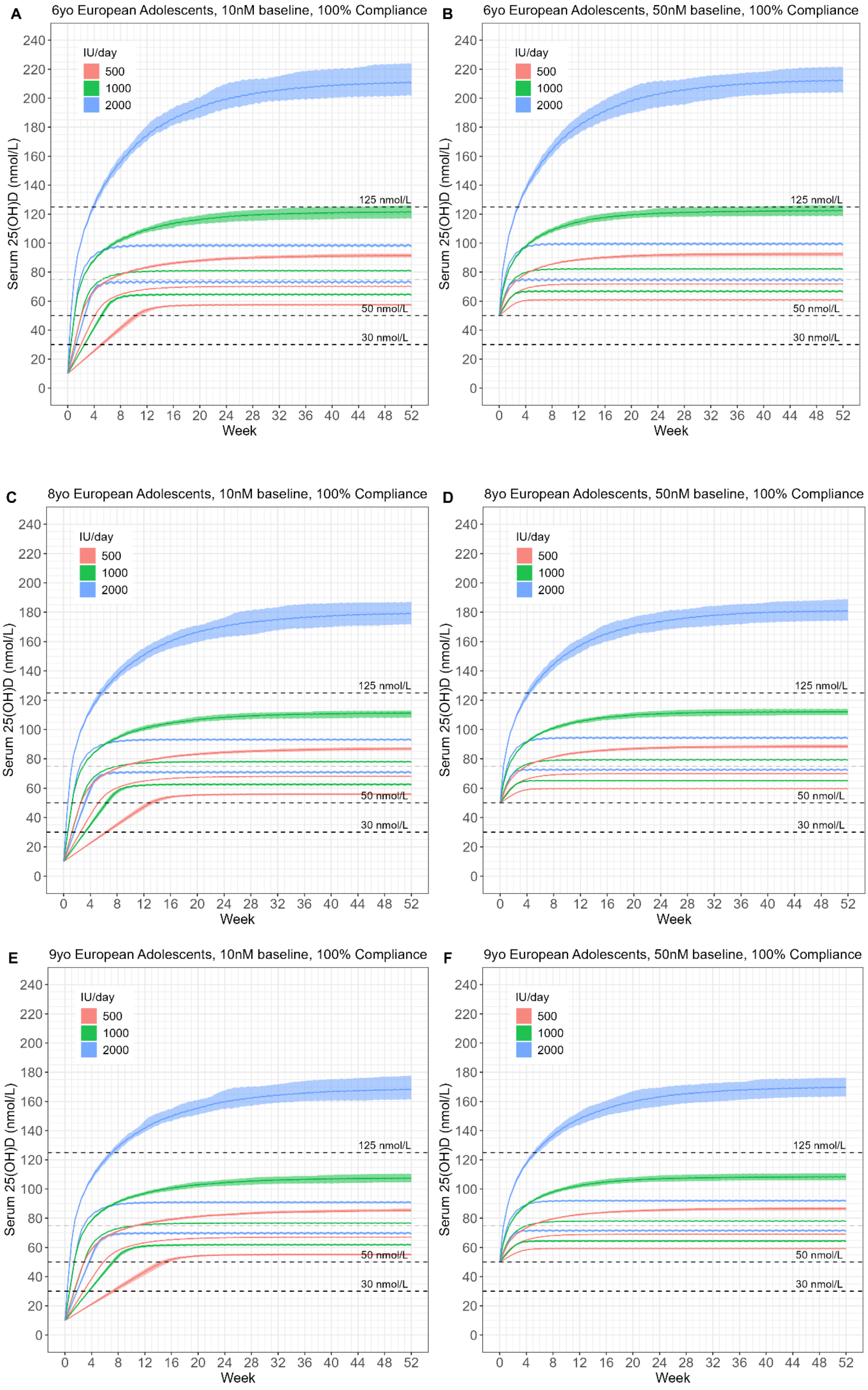
A
6yo European Adolescents, 10nM baseline, 100% Compliance
B
6yo European Adolescents, 50nM baseline, 100% Compliance
C
8yo European Adolescents, 10nM baseline, 100% Compliance
D
8yo European Adolescents, 50nM baseline, 100% Compliance
E
9yo European Adolescents, 10nM baseline, 100% Compliance
F
9yo European Adolescents, 50nM baseline, 100% Compliance
IU/day
500
1000
2000
125 nmol/L
50 nmol/L
30 nmol/L
Serum 25(OH)D (nmol/L)
0 20 40 60 80 100 120 140 160 180 200 220 240
Week
0 4 8 12 16 20 24 28 32 36 40 44 48 52

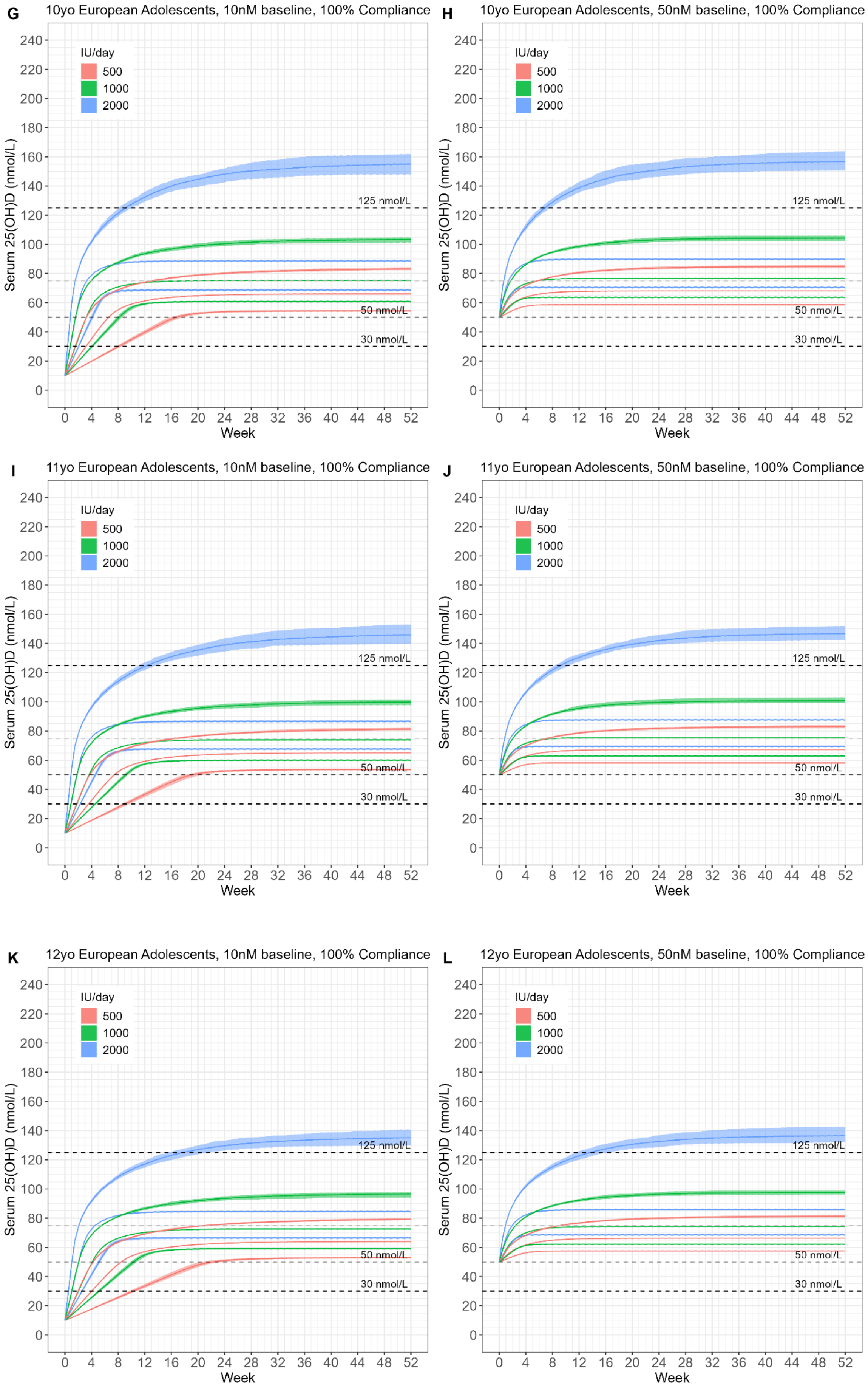
G
10yo European Adolescents, 10nM baseline, 100% Compliance
H
10yo European Adolescents, 50nM baseline, 100% Compliance
I
11yo European Adolescents, 10nM baseline, 100% Compliance
J
11yo European Adolescents, 50nM baseline, 100% Compliance
K
12yo European Adolescents, 10nM baseline, 100% Compliance
L
12yo European Adolescents, 50nM baseline, 100% Compliance
IU/day
500
1000
2000
Serum 25(OH)D (nmol/L)
Week
125 nmol/L
50 nmol/L
30 nmol/L

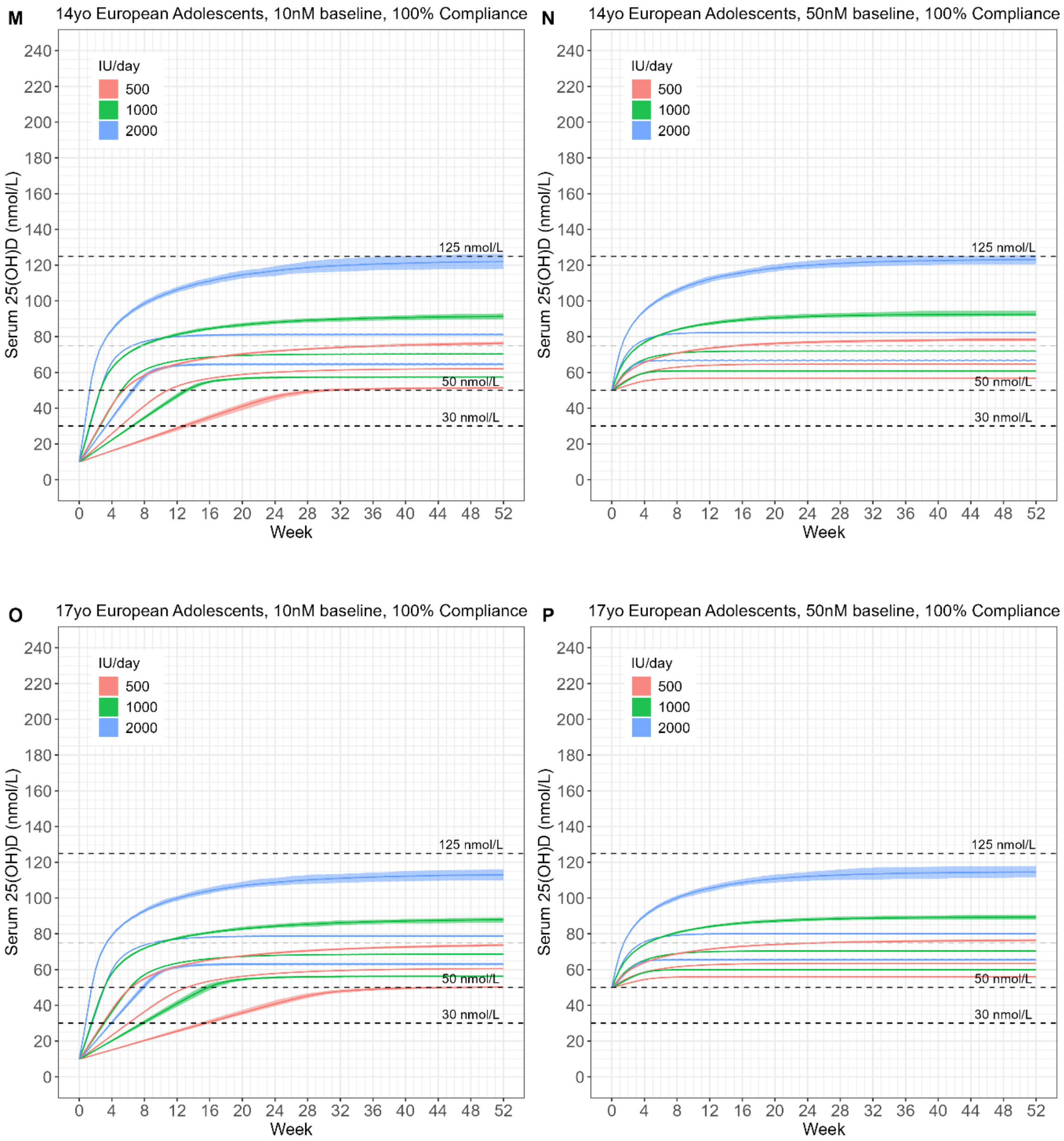

Figure S1. Simulation of serum 25(OH)D concentration for 1-year continuous daily dosing in the US and European children, aged 6 (A: 10 nmol/L baseline; B: 50 nmol/L baseline), 8 (C: 10 nmol/L baseline; D: 50 nmol/L baseline), 9 (E: 10 nmol/L baseline; F: 50 nmol/L baseline), 10 (G: 10 nmol/L baseline; H: 50 nmol/L baseline) and adolescents aged 11 (I: 10 nmol/L baseline; J: 50 nmol/L baseline), 12 (K: 10 nmol/L baseline; L: 50 nmol/L baseline), 14 (M: 10 nmol/L baseline; N: 50 nmol/L baseline) and 17 (O: 10 nmol/L baseline; P: 50 nmol/L baseline). The three lines mark the 2.5th, 50th and 97.5th percentiles with shaded areas showing the 95% confidence intervals.

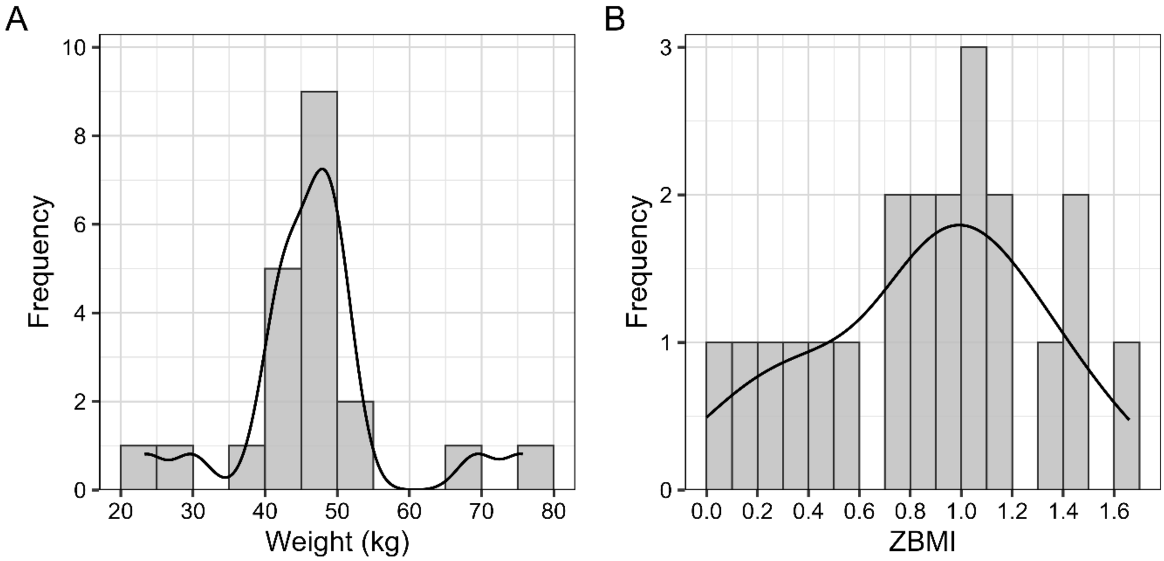

Figure S2. Distribution of weight (**A**) and ZBMI (**B**) in the external validation data. ZBMI: age-normalised body mass index. Black curve: kernel density estimate. The density estimates were calculated using a Gaussian kernel with an automatically selected bandwidth in the R package ggplot2.

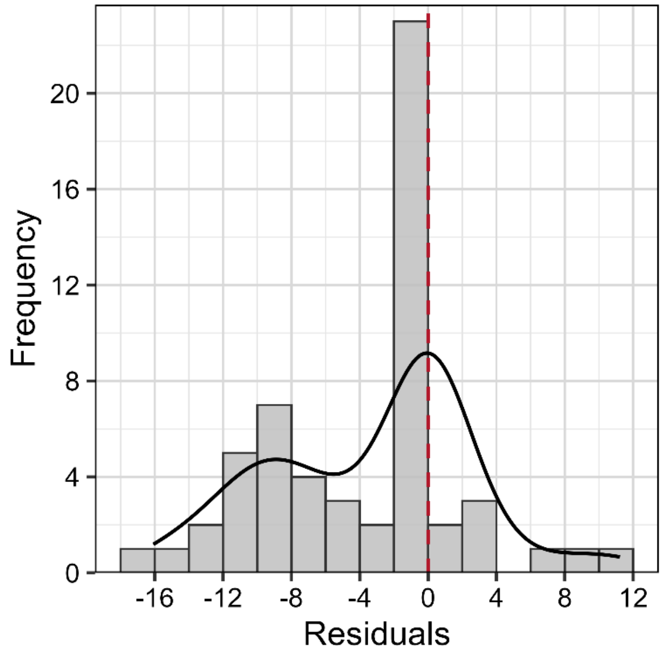

Figure S3. Residual distribution for the external validation of Model 1. Residuals: observations minus Model 1 predictions. The solid black curve: kernel density estimate. Dashed vertical red line: zero residual.

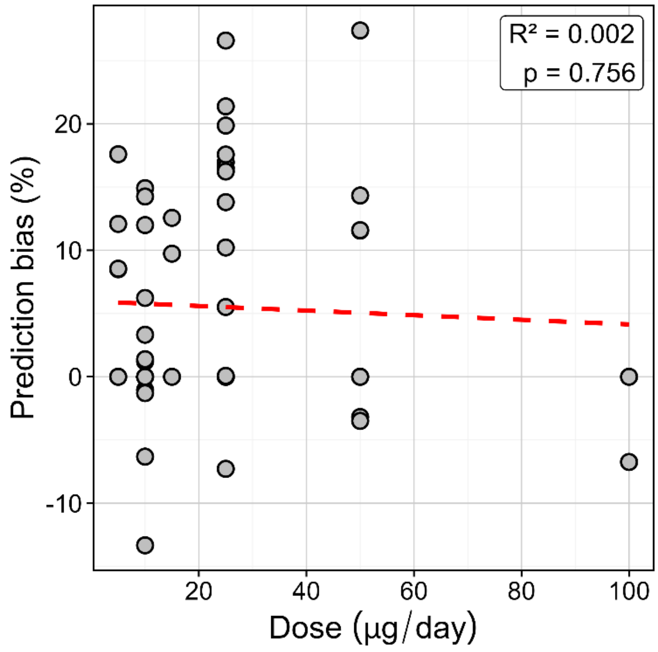

Figure S4. Prediction bias for Model 1 across doses. Prediction bias: the percentage difference between the prediction and observation of mean concentrations, based on the observations. Dashed red line: linear regression line. Legend: R^2 and the p-value for the slope.

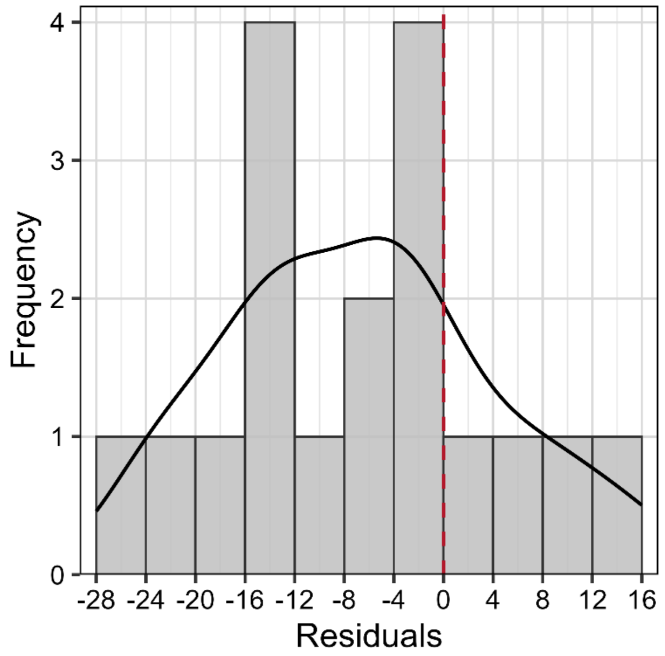

Figure S5. Residual distribution for the external validation of Model 2. The diagram is organised in the same way as Figure S3.

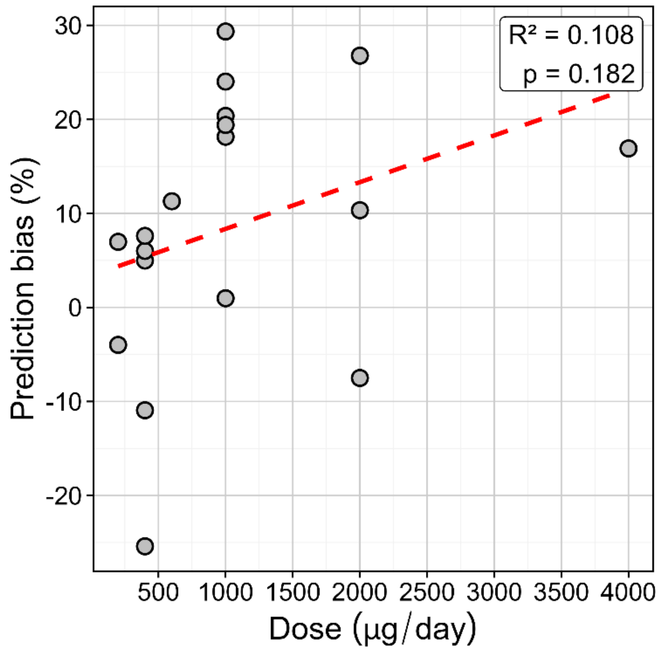

Figure S6. Prediction bias of Model 2 across doses. The diagram is organised in the same way as Figure S4.

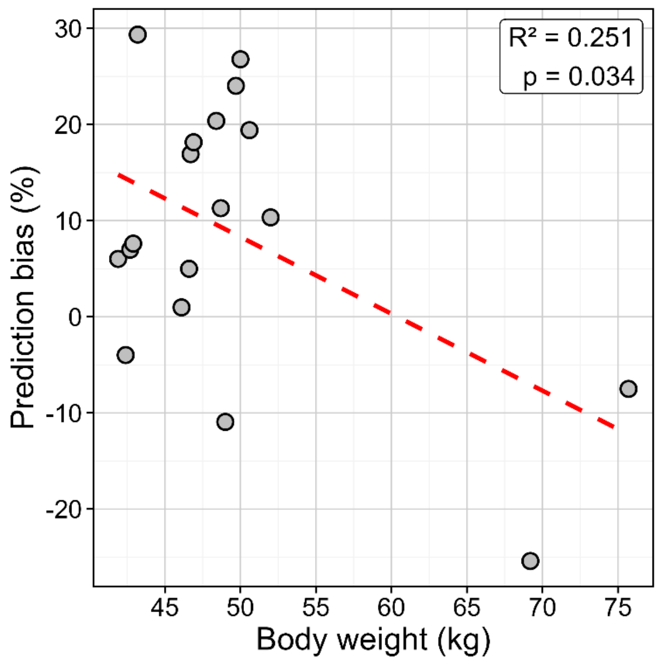

Figure S7. Prediction bias of Model 2 across body weights. The diagram is organised in the same way as Figure S4.

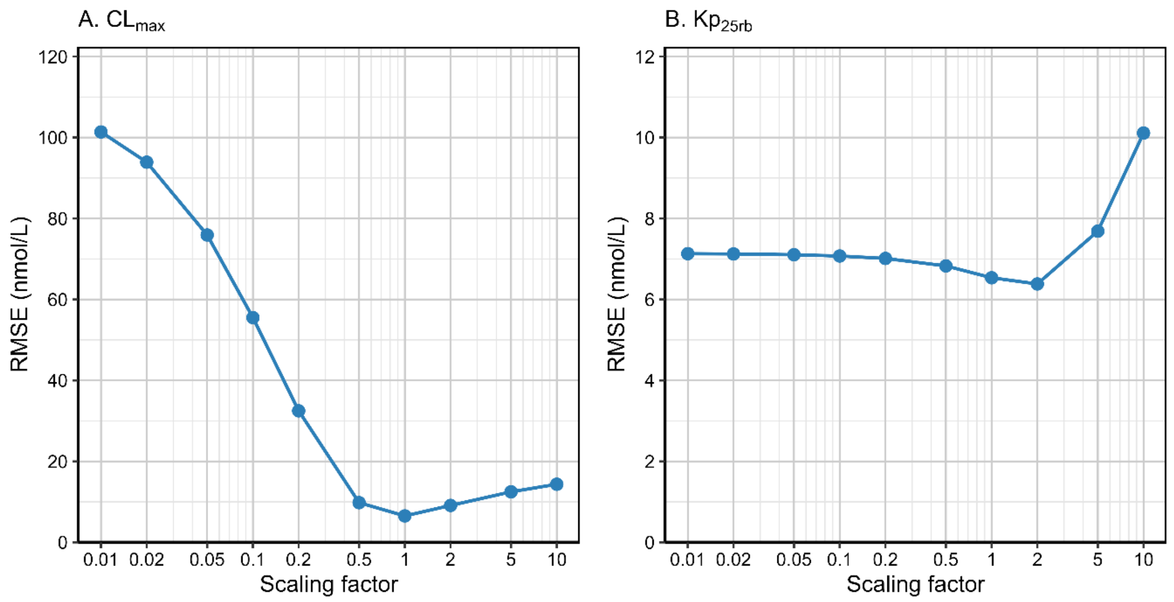

Figure S8. Sensitivity analysis of Model 3 performance to changes in CL_{max} and Kp_{25rb}. The parameters CL_{max} (**A**) and Kp_{25rb} (**B**) were varied singly from 0.01- to 10-fold of their nominal values, with all other parameters fixed at the nominal values. Root mean square error (RMSE) is plotted against each parameter which is in logarithmic scale.

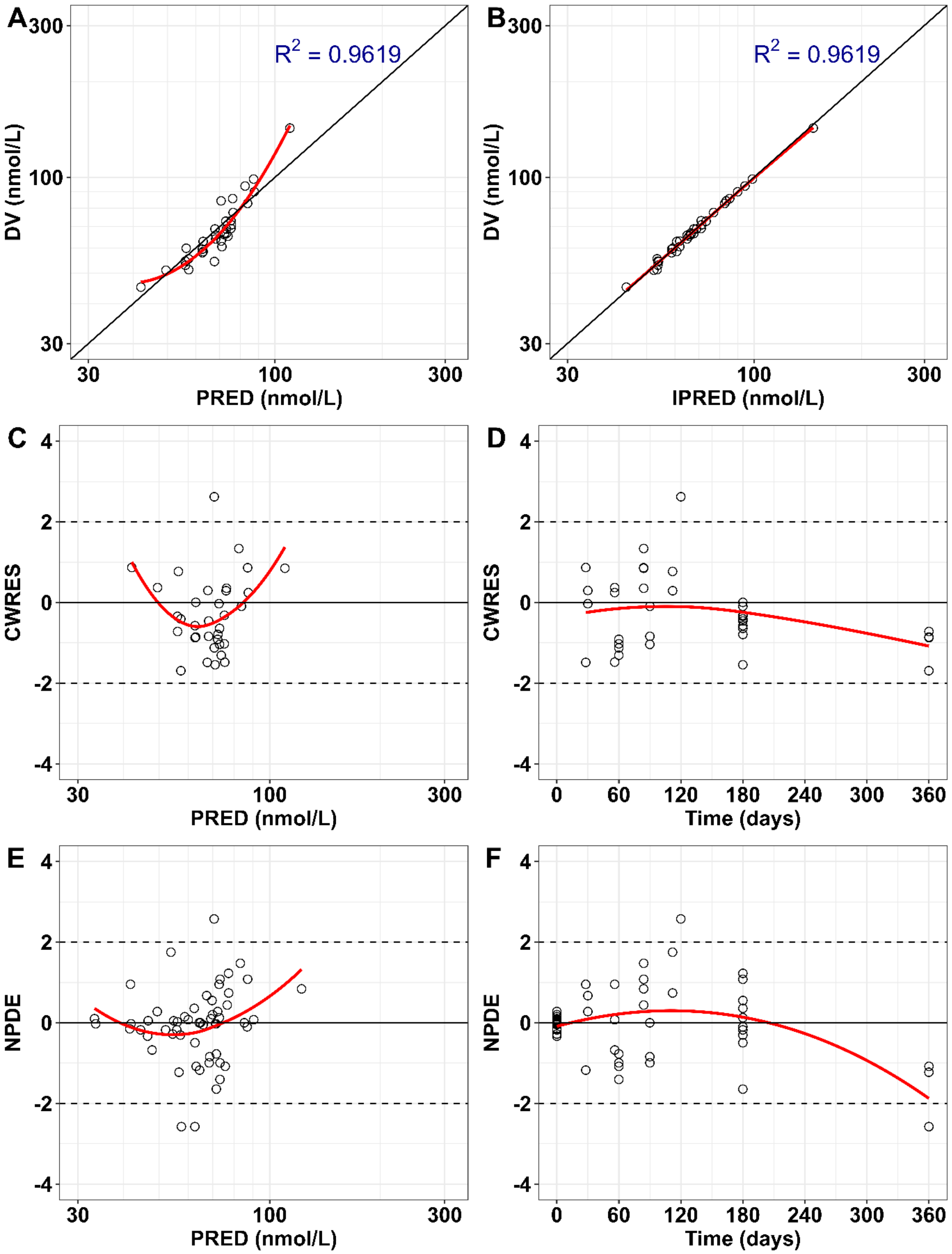

Figure S9. Diagnostic plots for Model 3. Goodness of fit: observations (DV in panels A and B) are plotted against the population (**A**) and individual (**B**) predictions. Conditional weighted residuals (CWRES) are plotted against population predictions (**C**) and time (**D**). Normalized prediction distribution errors (NPDEs) are plotted against population predictions (**E**) and time (**F**). Red curves: LOESS regression with α=5/3. PRED, population predictions. IPRED: individual predictions. DV: observations.

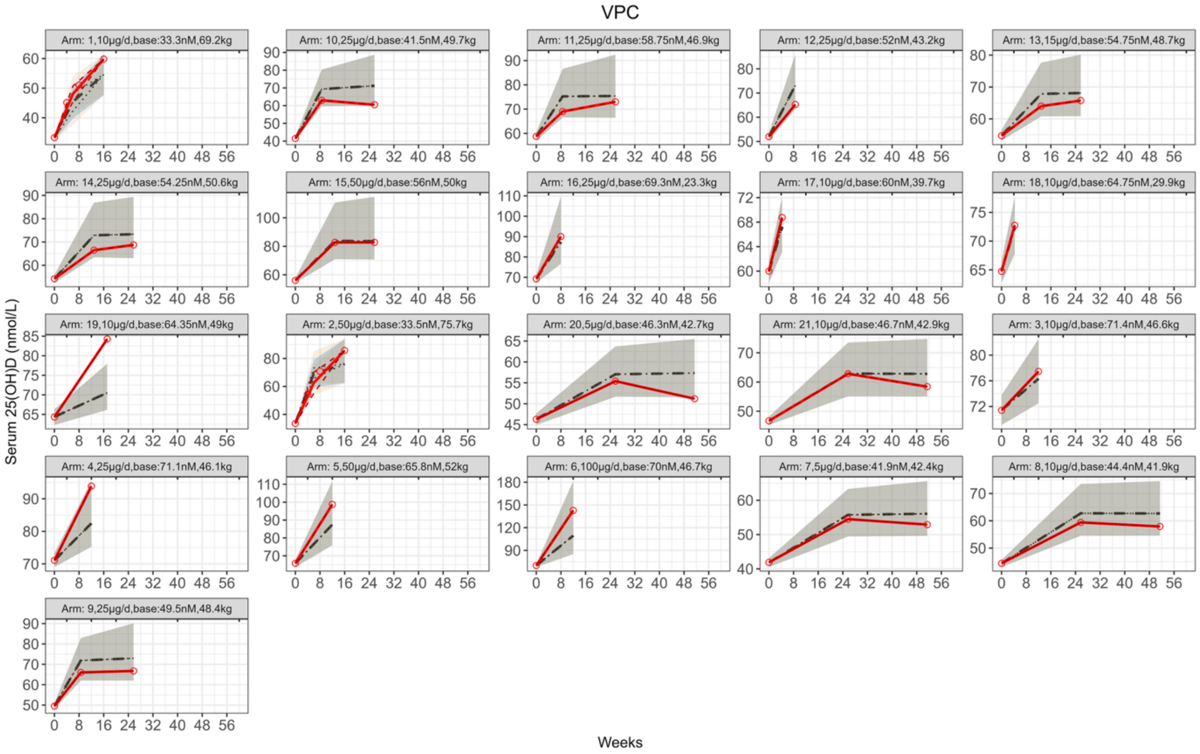

Figure S10. Visual predictive check for Model 3 fitting. Red points: observed mean values. Black dashed lines: predicted mean. Grey shaded band: 90% prediction interval. Dose ranges from 10 to 100 µg/day.

Table S1. Baseline characteristics of the external validation dataset.

Ref	Trial	Arm	DOSE	25(OH)D	DURATION	AGE	N	SEX	M (%)	F (%)	BMI	BMI percentile	Reported ZBMI	Weight (kg)	Height (cm)	Season	Race	ZBMI
1	1	1	10	33.3	112	16.3	22	M	68.2	31.8	23.5	61.6	NA	69.2	171.6	Late winter to spring	Black (African-American)	0.83
1	1	2	50	33.5	112	16.5	23	F	43.5	56.5	27.27	67.8	NA	75.7	166.6	Late winter to spring	Black (African-American)	1.41
2	2	3	10	71.4	84	11.3	59	M	NA	NA	20.44	67.6	NA	46.6	151	Winter	Black and White	1.05
2	2	4	25	71.1	84	11.1	63	M	NA	NA	20.76	70.4	NA	46.1	149	Winter	Black and White	1.16
2	2	5	50	65.8	84	11.4	61	M	NA	NA	22.21	71.5	NA	52	153	Winter	Black and White	1.42
2	2	6	100	70	84	11.5	58	M	NA	NA	20.76	67.4	NA	46.7	150	Winter	Black and White	1.09
3	3	7	5	41.9	360	11.4	73	F	0	100.0	18.6	NA	NA	42.4	151	All year round	White	0.33
3	3	8	10	44.4	360	11.4	74	F	0	100.0	18.52	NA	NA	41.9	150.4	All year round	White	0.30
4	4	9	25	49.5	180	11.2	78	F	42.3	57.7	21.6	NA	NA	48.4	148.1	Winter	Black and White	1.16
4	4	10	25	41.5	180	11.1	42	F	42.9	57.1	22.3	NA	NA	49.7	147.8	Winter	Black	1.32
4	4	11	25	58.75	180	11.3	36	F	41.7	58.3	20.7	NA	NA	46.9	148.5	Winter	White	0.95
5	5	12	25	52	180	11.3	51	F	47.1	52.9	20.6	NA	NA	43.2	148.4	Winter and spring	Black and White	0.92
6	6	13	15	54.75	180	11.7	207	F	47.3	52.7	21.3	NA	0.78	48.7	149.5	All year round	White	0.78
6	6	14	25	54.25	180	11.7	190	M	53.2	46.8	21.8	NA	0.82	50.6	150.9	All year round	White	0.82
6	6	15	50	56	180	11.7	207	F	47.3	52.7	21.6	NA	0.79	50	151.1	All year round	White	0.79
7	7	16	25	69.25	56	6.7	32	M	NA	NA	NA	NA	0	23.3	NA	NA	NA	0
8	8	17	10	60	30	8.9	41	M	65.9	34.1	21	NA	NA	39.7	136.2	Winter and early Spring	Black (African-American)	1.66
9	9	18	10	64.75	30	8.8	20	M	60.0	40.0	16.3	NA	NA	29.9	134.9	Winter and early Spring	Black (African-American)	0.13
10	10	19	10	64.35	120	13.3	10	F	40.0	60.0	22.99	63	NA	49	146	Winter and Spring	NA	1.08
11	11	20	5	46.3	360	11.4	65	F	0	100.0	19.05	NA	NA	42.7	149.7	All year round	NA	0.48
11	11	21	10	46.7	360	11.5	74	F	0	100.0	19.3	NA	NA	42.9	149.1	All year round	NA	0.53

Table S2. References for the external validation dataset.

Ref.	Reference
1	Dong Y, Stallmann-Jorgensen IS, Pollock NK, Harris RA, Keeton D, Huang Y, et al. A 16-Week Randomized Clinical Trial of 2000 International Units Daily Vitamin D_3 Supplementation in Black Youth: 25-Hydroxyvitamin D, Adiposity, and Arterial Stiffness. Journal of Clinical Endocrinology & Metabolism. 2010;95(10):4584-91.
2	Lewis RD, Laing EM, Hill Gallant KM, Hall DB, McCabe GP, Hausman DB, et al. A randomized trial of vitamin D_3 supplementation in children: dose-response effects on vitamin D metabolites and calcium absorption. Journal of Clinical Endocrinology & Metabolism. 2013;98(12):4816-25.
3	Mølgaard C, Larnkjaer A, Cashman KD, Lamberg-Allardt C, Jakobsen J, Michaelsen KF. Does vitamin D supplementation of healthy Danish Caucasian girls affect bone turnover and bone mineralization? Bone. 2010;46(2):432-9.
4	Rajakumar K, Moore CG, Yabes J, Olabopo F, Haralam MA, Comer D, et al. Effect of Vitamin D_3 Supplementation in Black and in White Children: A Randomized, Placebo-Controlled Trial. Journal of Clinical Endocrinology & Metabolism. 2015;100(8):3183-92.
5	Rajakumar K, Moore CG, Yabes J, Olabopo F, Haralam MA, Comer D, et al. Estimations of dietary vitamin D requirements in black and white children. Pediatric Research. 2016;80(1):14-20.
6	Sacheck JM, Van Rompay MI, Chomitz VR, Economos CD, Eliasziw M, Goodman E, et al. Impact of Three Doses of Vitamin D_3 on Serum 25(OH)D Deficiency and Insufficiency in At-Risk Schoolchildren. Journal of Clinical Endocrinology & Metabolism. 2017;102(12):4496-505.
7	Abrams SA, Hawthorne KM, Chen Z. Supplementation with 1000 IU vitamin D/d leads to parathyroid hormone suppression, but not increased fractional calcium absorption, in 4–8-y-old children: a double-blind randomized controlled trial1234. The American Journal of Clinical Nutrition. 2013;97(1):217-23.
8	Rajakumar K, Fernstrom JD, Janosky JE, Greenspan SL. Vitamin D insufficiency in preadolescent African-American children. Clinical pediatrics. 2005;44(8):683-92.
9	Rajakumar K, Fernstrom JD, Holick MF, Janosky JE, Greenspan SL. Vitamin D status and response to Vitamin $D_{(3)}$ in obese vs. non-obese African American children. Obesity (Silver Spring). 2008;16(1):90-5.
10	Cosenza L, Pezzella V, Nocerino R, Di Costanzo M, Coruzzo A, Passariello A, et al. Calcium and vitamin D intakes in children: a randomized controlled trial. BMC Pediatrics. 2013;13:86.
11	Viljakainen HT, Natri AM, Kärkkäinen M, Huttunen MM, Palssa A, Jakobsen J, et al. A positive dose-response effect of vitamin D supplementation on site-specific bone mineral augmentation in adolescent girls: a double-blinded randomized placebo-controlled 1-year intervention. Journal of Bone and Mineral Research. 2006;21(6):836-44.

Table S3. References for daily vitamin D doses of 2000 IU (50 µg) and 4000 IU (100 µg) in healthy adults.

Ref.	Reference
1	Chausmer, A. B. (2012). Dose Response to Vitamin D Supplementation in Postmenopausal Women. Annals of Internal Medicine, 157(5), 378.
2	Yao, P., Lu, L., Hu, Y., Liu, G., Chen, X., Sun, L., … Lin, X. (2015). A dose–response study of vitamin D3 supplementation in healthy Chinese: a 5-arm randomized, placebo-controlled trial. European Journal of Nutrition, 55(1), 383–392.
3	MacLennan, W. J., & Hamilton, J. C. (1977). Vitamin D supplements and 25-hydroxy vitamin D concentrations in the elderly. BMJ, 2(6091), 859–861. doi:10.1136/bmj.2.6091.860
4	Schwartz, J. B., Kane, L., & Bikle, D. (2016). Response of Vitamin D Concentration to Vitamin D3Administration in Older Adults without Sun Exposure: A Randomized Double-Blind Trial. Journal of the American Geriatrics Society, 64(1), 65–72.
5	Vieth R, Chan PC, MacFarlane GD. Efficacy and safety of vitamin D3 intake exceeding the lowest observed adverse effect level. Am J Clin Nutr 2001;73:288 –94.
6	Ng, K., Scott, J. B., Drake, B. F., Chan, A. T., Hollis, B. W., Chandler, P. D., … Fuchs, C. S. (2013). Dose response to vitamin D supplementation in African Americans: results of a 4-arm, randomized, placebo-controlled trial. The American Journal of Clinical Nutrition, 99(3), 587–598.
7	Himmelstein, S., Clemens, T. L., Rubin, A., & Lindsay, R. (1990). Vitamin D supplementation in elderly nursing home residents increases 25(OH)D but not 1,25(OH)2D. The American Journal of Clinical Nutrition, 52(4), 701–706.
8	LI-NG, M., ALOIA, J. F., POLLACK, S., CUNHA, B. A., MIKHAIL, M., YEH, J., & BERBARI, N. (2009). A randomized controlled trial of vitamin D3 supplementation for the prevention of symptomatic upper respiratory tract infections. Epidemiology and Infection, 137(10), 1396.
9	Cherniack, E. P., Florez, H. J., Hollis, B. W., Roos, B. A., Troen, B. R., & Levis, S. (2011). The Response of Elderly Veterans to Daily Vitamin D3 Supplementation of 2,000 IU: A Pilot Efficacy Study. Journal of the American Geriatrics Society, 59(2), 286–290.
10	Diamond, T., Wong, Y. K., & Golombick, T. (2012). Effect of oral cholecalciferol 2,000 versus 5,000 IU on serum vitamin D, PTH, bone and muscle strength in patients with vitamin D deficiency. Osteoporosis International, 24(3), 1101–1105.
11	Lehmann, U., Hirche, F., Stangl, G. I., Hinz, K., Westphal, S., & Dierkes, J. (2013). Bioavailability of Vitamin D2and D3in Healthy Volunteers, a Randomized Placebo-Controlled Trial. The Journal of Clinical Endocrinology & Metabolism, 98(11), 4339–4345.
12	Schroten, N. F., Ruifrok, W. P. T., Kleijn, L., Dokter, M. M., Silljé, H. H., Lambers Heerspink, H. J., … de Boer, R. A. (2013). Short-term vitamin D3 supplementation lowers plasma renin activity in patients with stable chronic heart failure: An open-label, blinded end point, randomized prospective trial (VitD-CHF trial). American Heart Journal, 166(2), 357–364.e2.
13	Mason, C., Xiao, L., Imayama, I., Duggan, C., Wang, C.-Y., Korde, L., & McTiernan, A. (2014). Vitamin D3 supplementation during weight loss: a double-blind randomized controlled trial. The American Journal of Clinical Nutrition, 99(5), 1015–1025.
14	Aranow, C., Kamen, D. L., Dall'Era, M., Massarotti, E. M., Mackay, M. C., Koumpouras, F., ... & Diamond, B. (2015). Randomized, double-blind, placebo-controlled trial of the effect of vitamin D3 on the interferon signature in patients with systemic lupus erythematosus. Arthritis & rheumatology, 67(7), 1848-1857.

15	Shieh, A., Ma, C., Chun, R. F., Witzel, S., Rafison, B., Contreras, H. T. M., … Adams, J. S. (2017). Effects of Cholecalciferol vs Calcifediol on Total and Free 25-Hydroxyvitamin D and Parathyroid Hormone. The Journal of Clinical Endocrinology & Metabolism, 102(4), 1133–1140.
16	Trang, H. M., Cole, D. E., Rubin, L. A., Pierratos, A., Siu, S., & Vieth, R. (1998). Evidence that vitamin D3 increases serum 25-hydroxyvitamin D more efficiently than does vitamin D2. The American Journal of Clinical Nutrition, 68(4), 854–858.
17	Fu, L., Yun, F., Oczak, M., Wong, B. Y. L., Vieth, R., & Cole, D. E. C. (2009). Common genetic variants of the vitamin D binding protein (DBP) predict differences in response of serum 25-hydroxyvitamin D [25(OH)D] to vitamin D supplementation. Clinical Biochemistry, 42(10-11), 1174–1177.
18	Von Hurst, P. R., Stonehouse, W., & Coad, J. (2009). Vitamin D supplementation reduces insulin resistance in South Asian women living in New Zealand who are insulin resistant and vitamin D deficient – a randomised, placebo-controlled trial. British Journal of Nutrition, 103(04), 549.
19	Garrett-Mayer, E., Wagner, C. L., Hollis, B. W., Kindy, M. S., & Gattoni-Celli, S. (2012). Vitamin D3 supplementation (4000 IU/d for 1 y) eliminates differences in circulating 25-hydroxyvitamin D between African American and white men. The American Journal of Clinical Nutrition, 96(2), 332–336.
20	Hata, T. R., Audish, D., Kotol, P., Coda, A., Kabigting, F., Miller, J., … Gallo, R. L. (2013). A randomized controlled double-blind investigation of the effects of vitamin D dietary supplementation in subjects with atopic dermatitis. Journal of the European Academy of Dermatology and Venereology, 28(6), 781–789.
21	Overton, E. T., Chan, E. S., Brown, T. T., Tebas, P., McComsey, G. A., Melbourne, K. M., ... & Yin, M. T. (2014, March). High-dose vitamin D and calcium attenuates bone loss with ART initiation: results from ACTG A5280. In Conference on Retroviruses and Opportunistic Infections.

Reference 50 µg/d: 2, 3, 4, 6, 7, 8, 9, 10, 11, 12, 13, 14.
Reference 100 µg/d: 1, 4, 5, 6, 14, 16, 17, 18, 19, 20, 21.

Table S4. Model parameters.

Model		Est. (natural log)	SE (natural log)	%RSE	Linear scale (95% CI)	IIV CV%[a]	Shrink%
1 *	Kp_{25rb}	-1.28	0.243	19	0.279 (0.173, 0.449)	114	26.1
	CL_{max}	3.07	0.13	4.22	0.0463 (0.0359, 0.0597)	69.1	17.6
	Additive error				0.000741		
	Proportional error				0.0459		
2 **	Kp_{25fm}	1.54	0.136	8.8	4.66 (3.6, 6.11)		
	Cl_{max}	-4.43	0.0787	1.78	0.0119 (0.0102, 0.0139)	62.8	13.1
	Additive error				0.00249		
	Proportional error				0.109		
3 ***	Kp_{25rb}	-1.22	0.336	27.6	0.296 (0.153, 0.571)	61.5	43.6
	CL_{max}	-3.32	0.138	4.17	0.0362 (0.0276, 0.0474)	66.6	2.29
	Additive error				0.00394		
	Proportional error				0.0186		

* The variance-covariance matrix for Model 1:

	η_{Kp25rb}	η_{CLmax}
η_{Kp25rb}	0.830	-0.116
η_{CLmax}	-0.116	0.391

** Model 2 assumes the partition coefficient of the lean mass Kp_{25lm} = 4.

*** The variance-covariance matrix for Model 3:

	η_{Kp25rb}	η_{CLmax}
η_{Kp25rb}	0.320	-0.178
η_{CLmax}	-0.178	0.367
